# Machine learning–driven integration of 24-hour ambulatory blood pressure and its variability

**DOI:** 10.1371/journal.pdig.0001499

**Published:** 2026-07-16

**Authors:** Evangelos Ntalianis, Everton Jose Santana, Nicholas Cauwenberghs, Veronique Cornelissen, Tatiana Kuznetsova

**Affiliations:** 1 Department of Cardiovascular Sciences, Research Unit Hypertension and Cardiovascular Epidemiology, KU Leuven, Leuven, Belgium; 2 Department of Rehabilitation Sciences, Rehabilitation in Internal Disorders, KU Leuven, Leuven, Belgium; Liverpool John Moores University - City Campus: Liverpool John Moores University, UNITED KINGDOM OF GREAT BRITAIN AND NORTHERN IRELAND

## Abstract

Twenty-four hour ambulatory blood pressure (BP) monitoring (24-hour ABPM) is considered the best out-of-office BP measurement to assess hypertension. Yet, the complexity of the obtained data, hinders their interpretation. Therefore, in this study, we investigated whether a clustering algorithm could facilitate the integrative interpretation of 24-hour ambulatory BP and its variability to construct distinctive clusters associated with cardiovascular (CV) outcomes. In 1344 community-dwelling individuals (mean age, 46.9 years; 50.6% women), we acquired 24-hour ABPM recordings, clinical data at baseline and we collected adverse outcomes (median follow-up time 19.4 years). We used dynamic time warping combined with the k-medoids clustering algorithm on the heart rate, systolic and diastolic BP recordings. Clinical characteristics and CV outcome were used to evaluate the validity of the derived clusters using the original cohort, while an external general population cohort (n = 1219) was used for further validation. We separated the dataset into 4 clusters. Cluster 1 showed the most favourable CV risk profile comprising younger individuals with the lowest 24-hour ABPM patterns. On the contrary, cluster 4 revealed the worst CV profile with older participants, higher office and 24-hour ABPM, higher medication intake and the highest BP variability. After adjusting for traditional risk factors, including office systolic BP cluster 4 had significantly higher risk of adverse CV events compared to cluster 1 (HR: 1.63; 95%CI:1.15-2.32; P = 0.006). Similar findings were made in the external validation cohort. Time series clustering of ABPM recordings may facilitate the integrative interpretation of 24-hour ambulatory BP and BP variability.

## Author summary

Twenty-four hour ambulatory blood pressure monitoring (24-hour ABPM) is considered the best out-of-office blood pressure measurement to identify white-coat or masked hypertension. Current clinical practice relies mostly on analysing the average 24-hour blood pressure (BP) values or the average day-time and night-time values separately. At the same time, in literature, an abundance of blood pressure variability (BPV) has emerged as independent predictors of adverse cardiovascular events. However, the large number of BPV indices makes their interpretation and integration with the average 24-hour ABPM value more challenging. Therefore, we investigated if using a multivariable unsupervised machine learning model we could derive clinically meaningful groups integrating both the average BP values and the BPV into a single model. By combining these features, we aimed to facilitate the use and interpretation of this otherwise complex time-series data. Using the raw 24-hour systolic and diastolic BP and heart rate time series recordings, we extracted 4 distinct profiles of 24-hour ABPM and BPV associated with different cardiovascular risk. Notably, our approach provided statistically significant additive information beyond the cardiovascular risk score that clinicians are currently using. Further testing our model in an external validation cohort showed similar results. Thus, time series clustering of ABPM recordings may facilitate the integrative interpretation of 24-hour ambulatory BP and BP variability.

## Introduction

Cardiovascular (CV) disease is the leading cause of death worldwide, accounting for an estimated 17.9 million deaths annually [1]. Blood pressure (BP) is one of the most important modifiable risk factor associated with adverse CV outcome [2]. As such, the accurate assessment of BP plays a key role for the prevention and management of CV diseases. Besides office BP measurements, ambulatory BP monitoring (ABPM) has emerged as a valuable tool to assess BP and especially to detect white-coat, masked and sustained hypertension [3,4].

Twenty-four hour ABPM is the most effective out-of-office approach for assessing both average BP and short-term changes in BP and heart rate [5]. As such, it paved the way to investigate BP variability (BPV), which captures short-term BP fluctuations representing the dynamic nature of 24-hour ABPM patterns [6]. Several studies reported BPV as an independent predictor of adverse events, proposing a variety of indices including the standard deviation of the 24-hour BP readings [7,8], non-dipping patterns, [9,10] and the morning surge [11].

Current clinical practice relies mostly on the average value of either the whole 24-hour ambulatory BP or on the average value during day- and night-time [12]. This approach neglects the high dimensionality of ABPM recordings and the true value of the multivariate time series data. Furthermore, the plethora of BPV indices that can be extracted from ABPM recordings complicates analysis and interpretation. [13] Therefore, there is an unmet need for advanced computational methods that can capture the complex interdependencies within ABPM data and establish an integrative and clinically valuable interpretation.

Towards this direction, machine learning (ML) offers a promising alternative for analysing complex ABPM data. ML algorithms suitable for time series data can capture non-linear patterns across 24-hour ABPM recordings, potentially revealing insights beyond known BPV indices. Yet, to our knowledge, the application of ML algorithms on raw ABPM recordings has not been explored. Hence, in this study, we investigated whether the utilisation of unsupervised ML to raw ABPM recordings enables a more integrated assessment of ABPM variables, including BPV with the goal to identify distinct BP clusters associated with baseline and future CV risk.

## Materials and methods

### Study participants

For this study we used 24-hour ABPM recordings from the Flemish Study on Environment, Genes and Health Outcomes (FLEMENGHO) [14,15] and from the European Project on Genes for Hypertension (EPOGH) [15,16]. Both cohorts comprised randomly recruited individuals from northeastern Belgium (FLEMENGHO) and from Italy, Poland and Russia (EPOGH). Both studies received ethical approval.

*Training cohort –* From 1989 to 2008, we obtained 24-hour ABPM recordings from 2904 FLEMENGHO participants. We excluded 1512 individuals due to missing BP measurements during night-time (n = 1376) or for more than three consecutive hours (n = 133; 131 during day-time, 2 during night-time). Three more were removed due to no available measurements. In addition, we removed from the dataset 48 more individuals due to missing clinical information. Thus, the final dataset consisted of 1344 time series 24-hour ABPM recordings.

*Validation cohort –* To assess the clinical significance of integrative ABPM interpretations, we applied the trained model on 24-hour ABPM recordings obtained from the EPOGH cohort between 1999 and 2008. Following the same exclusion criteria, the final dataset comprised 1219 individuals.

### BP measurements

As described elsewhere, [14,15] conventional BP was measured using a standard mercury sphygmomanometer, with participants seated and rested for at least two minutes. 24-hour ambulatory BP was recorded using validated devices in 20-minute intervals during day-time and in 45 minute intervals during night-time [14,15]. Average BP values for the 24-hour, day-time and night-time periods were weighted by the time interval between consecutive readings, measured in minutes. The database organization is described in detail in S1 Text.

Additionally, we calculated BPV indices, including the dispersion (24-hour and day-/night-time weighted), average real variability, time rate, instability (range, peak, through) and nocturnal fall, night/day ratio and morning surge. S1 Table summarises the formulae used to calculate the BPV indices. For calculating weighted dispersion, nocturnal fall, night/day ratio, and morning surge, the day and night periods were defined as [7 am -10 pm) and [10 pm - 7 am) respectively.

In-office hypertension, masked, white-coat and sustained hypertension were defined following the recent guidelines [12]. For detailed information on the definitions, see S2 Table.

### Outcome assessment

We assessed the occurrence of adverse CV events by collecting outcome data in both FLEMENGHO and EPOGH. In the FLEMENGHO cohort, we ascertained CV fatal events using the Belgian health registry. Non-fatal events were assessed through follow-up visits or via a telephone interview using a standardized questionnaire. Self-reported diseases were cross-checked and supplemented with medical reports from general practitioners and local hospitals. Similar procedures were followed in the EPOGH cohort. In both cohorts, CV events comprised coronary events (myocardial infarction, acute coronary syndrome, angina pectoris/ischemic heart disease requiring revascularization), heart failure, atrial fibrillation and pacemaker implantation along with fatal and non-fatal stroke, including transient ischaemic attack, and peripheral revascularisation. In cases of multiple events, only the first event was considered.

### Data processing and analysis

We separated the dataset into clusters of 24-hour ABPM by employing an unsupervised learning approach. The computational pipeline was developed using the standard Python 3.9 environment (www.python.org) (S1 Fig). All python scripts are available online at https://github.com/HCVE/24h_abpm_clustering.

*Data pre-processing*: First, we applied a quality control step on the ABPM data. We excluded ABPM recordings with periods of more than 3 consecutive hours between successful BP measurements. After that, we used the raw ABPM recordings without any signal processing.

*Model training*: To derive the desired clusters, we applied k-medoids clustering algorithm [17] combined with dynamic time warping [18] to the ABPM time series of 24-hour systolic (SBP) and diastolic blood pressure (DBP), and 24-hour heart rate. Mean arterial pressure (MAP) and pulse pressure (PP) were not considered due to their strong computational correlation with SBP and DBP. We used the raw time series recordings without performing any scaling (or standardization) to capture both temporal characteristics (dynamic fluctuations) of the 24-hour ABPM and the BP levels. DTW was calculated jointly for all variables using “Tslearn” library. It should be noted that the timestamps were not included as a separate variable and thus the recordings were considered equally spaced by index. To identify the optimal number of clusters, we calculated a hybrid cluster validity index (CVI) [19] as the square root of the product between the two individual CVIs, i.e., silhouette and Dunn index. Further details on dynamic time warping and the motivation behind selecting this computational approach are presented in S2 Text.

### Statistical analysis

The database was managed using SAS software version 9.4 (SAS Institute, Cary, NC, USA). Statistical analysis was performed using Python 3.9 and its dedicated library for statistics “statsmodels”. To evaluate the clinical relevance of the BP clusters, we compared the clinical characteristics, 24-hour ABPM summary and BPV indices across clusters in both the training cohort (FLEMENGHO) and the external validation cohort (EPOGH). We statistically compared the means of the continuous variables’ distributions using z-test, while to assess differences in the proportions of the continuous variables the 𝛘^2^ statistical test was used. In addition, we presented the cumulative incidence of CV events per cluster using Kaplan-Meier method. We calculated the hazard ratios (HRs) through Cox regression model. Any violations in the assumptions of the proportional hazard were evaluated using Schoenfeld residuals. The HRs were adjusted for traditional risk factors, namely age, sex, body mass index (BMI), office SBP, total cholesterol, smoking and drinking status, diabetes mellitus, a history of a CV disease, and intake of antihypertensive medication.

## Results

For model training, we used ABPM data of 1344 FLEMENGHO participants, comprising 709 (53%) women. The mean age at baseline was 46.9 ± 15.9 years and the mean BMI was 25.6 ± 4.4 kg/m^2^. Of the 427 individuals diagnosed with hypertension (30.7% of the cohort), 209 (48.9%) received antihypertensive treatment.

### 24-Hour ABPM clusters

**Fig 1:**
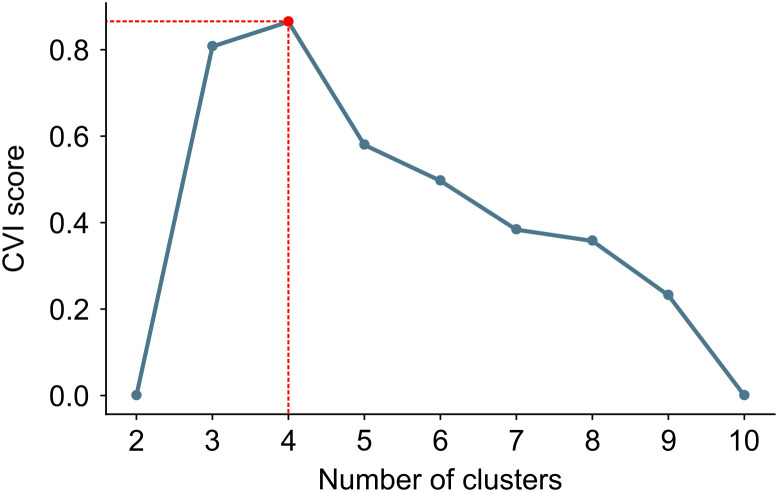
Cluster validity score (CVI) to estimate the optimal number of clusters. It was calculated as the square root of the product of silhouette score and Dunn index. Red line indicates the optimal number of clusters and the value selected to for the analysis.

The hybrid CVI score suggested that the participants should be divided into 4 clusters (Fig 1). The correctness of the number of clusters was further validated through cluster stability analysis which indicated that 4 cluster is the optimal number as well (S2 Fig). Detailed information on the clustering stability analysis are provided in S3 Text. As such, each 24-hour ABPM recording was assigned to one of the four clusters. Fig 1 presents the average profile of each cluster, including the profiles in 24-hour MBP and 24-hour PP. Clusters 1 and 2 had similar 24-hour SBP and DBP profiles, both within the normal range. However, they differed considerably in heart rate, with cluster 1 showing a higher 24-hour heart rate profile. On the other hand, cluster 4 presented the highest 24-hour SBP and DBP profiles, yet its HR profile closely resembled that of cluster 2. Cluster 3 showed intermediate 24-hour SBP levels, falling between clusters 1, 2, and 4, while its 24-hour DBP profile was similar to cluster 4. Of note, the 24-hour DBP profile of cluster 3 had the same day-time level but showed a higher decrease during night-time.

### 24-Hour ABPM and CV risk factors

Tables 1 and 2 show, respectively, the clinical characteristics and the 24-hour ABPM summary statistics of the FLEMENGHO participants by cluster. We observed significant differences between all clusters in age and office SBP and DBP (Table 1). Cluster 1 included predominantly female participants with the most favourable CV risk profile, with the lowest office BP and the lowest medication intake. On the other hand, cluster 4 showed the worst CV risk profile. Participants assigned to this cluster were mainly male of older age with higher office SBP and DBP and with higher medication intake. Clusters 2 and 3 showed intermediate values, falling between those of clusters 1 and 4.

**Table 1:** Clinical characteristics of FLEMENGHO participants by 24-hour ABPM cluster.

	Cluster 1 (n = 485)	Cluster 2 (n = 394)	Cluster 3 (n = 318)	Cluster 4 (n = 147)
**Anthropometrics**				
Females, n(%)	350 (72.16)	156 (39.59)*	131 (41.19)*	43 (29.25)*†‡
Age, years	43.07 ± 14.0	46.11 ± 17.2*	48.06 ± 13.91*	58.75 ± 14.88*†‡
Body mass index, kg/m²	24.71 ± 4.52	25.29 ± 4.4	26.44 ± 4.1*†	27.51 ± 3.86*†‡
Systolic pressure, mm Hg	116.49 ± 12.17	124.41 ± 14.51*	132.07 ± 15.53*†	148.47 ± 18.27*†‡
Diastolic pressure, mm Hg	72.35 ± 8.56	74.04 ± 9.42*	81.59 ± 9.62*†	83.8 ± 12.27*†‡
Heart rate, beats/min	75.37 ± 9.96	65.14 ± 7.95*	73.77 ± 10.11*†	66.55 ± 10.99*‡
**Questionnaire data**				
Drinking alcohol, n(%)	68 (14.02)	93 (23.6)*	76 (23.9)*	39 (26.53)*
Current smoking, n(%)	158 (32.58)	79 (20.05)*	115 (36.16)†	21 (14.29)*‡
History of stroke, n(%)	5 (1.03)	0 (0.0)*	5 (1.57)†	5 (3.4)*†
History of CV diseases, n(%)	33 (6.8)	33 (8.38)	20 (6.29)	32 (21.77)*†‡
History of DM, n(%)	10 (2.06)	10 (2.54)	12 (3.77)	9 (6.12)*†
**Medication**				
Diuretics, n(%)	13 (2.68)	35 (8.88)*	15 (4.72)†	29 (19.73)*†‡
Beta Blockers, n(%)	22 (4.54)	44 (11.17)*	12 (3.77)†	35 (23.81)*†‡
Angiotensin, n(%)	3 (0.62)	10 (2.54)*	7 (2.2)*	12 (8.16)*†‡
Calcium Channel Blocker, n(%)	3 (0.62)	13 (3.3)*	9 (2.83)*	20 (13.61)*†‡
Anti-hypertensive, n(%)	37 (7.63)	68 (17.26)*	32 (10.06)†	65 (44.22)*†‡
**Hypertension**				
Masked hypertension, n(%)	27 (5.57)	36 (9.14)*	97 (30.5)*†	21 (14.29)*‡
White coat hypertension, n(%)	17 (3.51)	27 (6.85)*	27 (8.49)*	1 (0.68)†‡
Sustained hypertension, n(%)	39 (8.04)	76 (19.29)*	102 (32.08)*†	122 (82.99)*†‡
**Biochemical data**				
Blood glucose, mmol/L	4.88 ± 0.87	5.07 ± 1.19*	4.98 ± 0.92	5.66 ± 2.47*†‡
Total cholesterol, mmol/L	5.48 ± 1.06	5.45 ± 1.14	5.73 ± 1.13*†	5.72 ± 1.06*†
HDL cholesterol, mmol/L	1.4 ± 0.30	1.35 ± 0.30*	1.27 ± 0.32*†	1.24 ± 0.32*†
LDL cholesterol, mmol/L	2.87 ± 0.42	2.91 ± 0.58	3.16 ± 0.46*†	3.38 ± 0.50*†‡
Serum creatinine, μmol/L	83.87 ± 14.56	93.43 ± 19.92*	91.18 ± 15.92*	99.31 ± 19.21*†‡

Values are mean (±SD) or number of subjects (%). Significance for between-cluster differences: *P < 0.05 vs Cluster 1; †P < 0.05 vs Cluster 2; ‡P < 0.05 vs Cluster 3. HDL, high density lipoprotein; LDL, low density lipoprotein.

**Table 2:** 24-Hour ambulatory blood pressure monitoring statistics by 24-hour ABPM cluster.

	Cluster 1 (n = 485)	Cluster 2 (n = 394)	Cluster 3 (n = 318)	Cluster 4 (n = 147)
**24-hour Systolic blood pressure**				
Mean 24-hour ABPM, mm Hg	111.74 ± 6.43	115.26 ± 7.53*	124.59 ± 7.23*†	138.01 ± 9.91*†‡
Mean day-time, mm Hg	115.94 ± 6.73	119.17 ± 7.76*	130.73 ± 7.26*†	142.55 ± 10.21*†‡
Mean night-time, mm Hg	104.56 ± 7.78	108.6 ± 9.07*	114.07 ± 9.17*†	130.29 ± 12.01*†‡
Day-night difference, mm Hg	11.39 ± 6.39	10.58 ± 7.08	16.66 ± 7.12*†	12.25 ± 9.51†‡
**24-hour Diastolic blood pressure**				
Mean 24-hour ABPM, mm Hg	67.51 ± 4.76	67.7 ± 5.24	77.17 ± 5.55*†	79.53 ± 8.33*†‡
Mean day-time, mm Hg	71.62 ± 5.06	71.29 ± 5.68	82.79 ± 5.75*†	83.59 ± 8.43*†
Mean night-time, mm Hg	60.48 ± 5.8	61.59 ± 6.27*	67.53 ± 7.08*†	72.65 ± 9.35*†‡
Day-night difference, mm Hg	11.13 ± 5.01	9.7 ± 5.69*	15.26 ± 6.07*†	10.94 ± 5.8†‡
**24-hour Mean blood pressure**				
Mean 24-hour ABPM, mm Hg	81.47 ± 4.76	83.09 ± 5.36*	92.03 ± 5.84*†	98.45 ± 8.21*†‡
Mean day-time, mm Hg	85.61 ± 5.02	86.68 ± 5.69*	97.77 ± 5.99*†	102.55 ± 8.28*†‡
Mean night-time, mm Hg	74.39 ± 6.03	76.97 ± 6.58*	82.17 ± 7.45*†	91.49 ± 9.71*†‡
Day-night difference, mm Hg	11.22 ± 5.31	9.71 ± 5.75*	15.6 ± 6.15*†	11.06 ± 6.7†‡
**24-hour Pulse pressure**				
Mean 24-hour ABPM, mm Hg	44.23 ± 5.76	47.56 ± 6.49*	47.43 ± 5.91*	58.48 ± 9.48*†‡
Mean day-time, mm Hg	44.32 ± 6.11	47.89 ± 6.75*	47.94 ± 6.39*	58.96 ± 9.89*†‡
Mean night-time, mm Hg	44.07 ± 6.09	47.01 ± 6.97*	46.55 ± 6.24*	57.65 ± 10.24*†‡
Day-night difference, mm Hg	0.25 ± 4.17	0.88 ± 4.43*	1.39 ± 4.65*	1.31 ± 6.82*
**24-hour Pulse rate**				
Mean 24-hour ABPM, mm Hg	77.67 ± 6.91	63.63 ± 5.82*	76.42 ± 6.89*†	65.31 ± 8.59*†‡
Mean day-time, mm Hg	82.64 ± 7.63	66.74 ± 6.34*	81.87 ± 7.6†	68.38 ± 9.15*†‡
Mean night-time, mm Hg	69.17 ± 7.87	58.27 ± 7.07*	67.08 ± 7.84*†	60.05 ± 8.8*†‡
Day-night difference, mm Hg	13.46 ± 7.12	8.47 ± 6.53*	14.8 ± 7.13*†	8.33 ± 5.77*‡

Values are mean (±SD) or number of subjects (%). Significance for between-cluster differences: *P < 0.05 vs Cluster 1; †P < 0.05 vs Cluster 2; ‡P < 0.05 vs Cluster 3. ABPM, ambulatory blood pressure monitoring.

**Fig 2:**
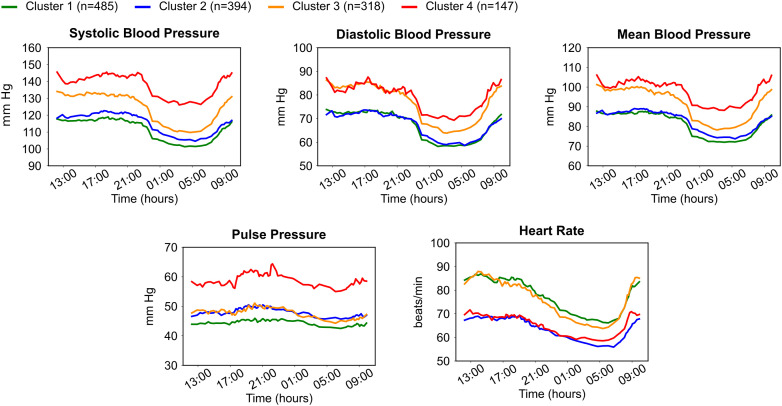
24-hour ABPM profiles by 24-hour ABPM cluster. Profiles were calculated as the average of the individual 24-hour ABPM traces assigned to each cluster.

**Fig 3:**
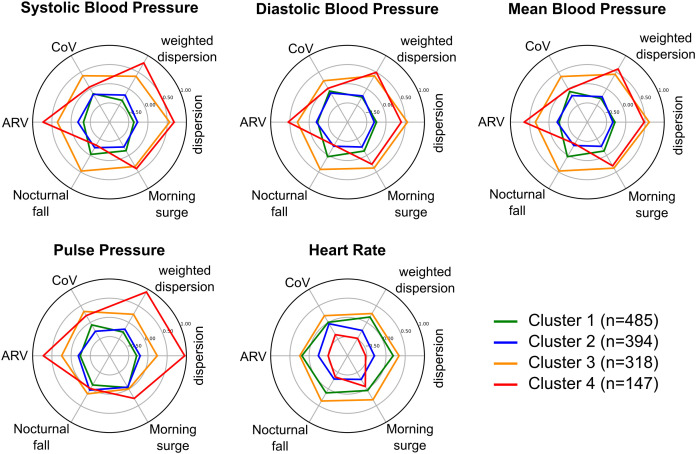
Z-score of blood pressure variability by k-medoids clusters in FLEMENGHO participants. Positive z-scores indicate higher values than the mean value of the FLEMENGHO cohort, while negative z-scores indicate lower values. CV represents coefficient of variation and ARV average real variability.

In addition, all clusters differed significantly in 24-hour SBP and DBP indices (Table 2). Notably, clusters 3 and 4 differed significantly in night-time DBP but they showed similar values in day-time, validating the observations from the 24-hour ABPM profiles (Fig 2). Comparing the BPV indices, we observed significant differences across all clusters, with cluster 4 consistently showing higher values (Fig 3, Table 3). Specifically, clusters 1 and 2 showed lower BP variability than the average variability observed in the population. However, they differed primarily between each other in the magnitude of nocturnal fall of DBP. On the other hand, clusters 3 and 4 revealed higher than average BP variability. Differentiating mostly in nocturnal fall, cluster 3 was characterised by significantly higher % nocturnal fall in both systolic and diastolic BP.

**Table 3:** Blood pressure variabilities of FLEMENGHO participants by k-medoids clusters.

	Cluster 1 (n = 485)	Cluster 2 (n = 394)	Cluster 3 (n = 318)	Cluster 4 (n = 147)
**Dispersion**				
SBP, mm Hg	11.46 ± 2.55	11.74 ± 2.72	14.38 ± 3.16*†	14.89 ± 3.46*†
DBP, mm Hg	10.59 ± 2.71	10.38 ± 2.52	13.09 ± 3.29*†	12.62 ± 3.76*†
MBP, mm Hg	10.68 ± 2.69	10.48 ± 2.47	13.35 ± 3.13*†	13.05 ± 3.21*†
PP, mm Hg	8.04 ± 2.09	8.23 ± 2.26	9.47 ± 2.46*†	11.49 ± 4.06*†‡
PR, beats/min	13.46 ± 5.1	10.76 ± 4.85*	14.33 ± 6.06*†	9.58 ± 4.38*†‡
**weighted Dispersion**				
SBP, mm Hg	10.05 ± 2.34	10.47 ± 2.48*	12.06 ± 2.95*†	13.37 ± 3.29*†‡
DBP, mm Hg	9.19 ± 2.6	9.25 ± 2.39	11.02 ± 3.07*†	11.37 ± 3.7*†
MBP, mm Hg	9.26 ± 2.51	9.38 ± 2.33	11.23 ± 2.93*†	11.79 ± 3.18*†
PP, mm Hg	7.84 ± 2.02	8.03 ± 2.23	9.24 ± 2.47*†	11.11 ± 3.98*†‡
PR, beats/min	11.52 ± 4.53	9.56 ± 4.37*	12.08 ± 5.59†	8.45 ± 3.89*†‡
**Coefficient of Variation**				
SBP, mean (SD)	10.14 ± 2.21	10.08 ± 2.27	11.41 ± 2.56*†	10.69 ± 2.35*†‡
DBP, mean (SD)	15.5 ± 4.06	15.21 ± 3.75	16.75 ± 4.37*†	15.84 ± 4.87‡
MBP, mean (SD)	12.94 ± 3.23	12.5 ± 2.9*	14.34 ± 3.5*†	13.18 ± 3.23†‡
PP, mean (SD)	18.08 ± 3.91	17.16 ± 4.0*	19.81 ± 4.55*†	19.37 ± 5.36*†
PR, mean (SD)	16.97 ± 6.04	16.67 ± 7.27	18.38 ± 7.48*†	14.5 ± 6.55*†‡
**Average real variability**				
SBP, mm Hg	8.86 ± 2.01	9.16 ± 2.07*	10.49 ± 2.53*†	11.47 ± 2.85*†‡
DBP, mm Hg	8.07 ± 2.32	8.13 ± 2.08	9.41 ± 2.59*†	10.04 ± 3.45*†‡
MBP, mm Hg	8.2 ± 2.19	8.29 ± 2.07	9.7 ± 2.59*†	10.51 ± 3.08*†‡
PP, mm Hg	8.08 ± 2.18	8.17 ± 2.38	9.37 ± 2.63*†	10.79 ± 4.14*†‡
PR, beats/min	8.66 ± 3.39	7.2 ± 2.85*	8.91 ± 3.96†	6.34 ± 2.41*†‡
**Time rate**				
SBP, mm Hg/min	0.37 ± 0.14	0.39 ± 0.19	0.45 ± 0.15*†	0.5 ± 0.25*†‡
DBP, mm Hg/min	0.34 ± 0.15	0.34 ± 0.16	0.4 ± 0.14*†	0.43 ± 0.21*†
MBP, mm Hg/min	0.35 ± 0.15	0.35 ± 0.17	0.42 ± 0.15*†	0.46 ± 0.2*†‡
PP, mm Hg/min	0.35 ± 0.13	0.36 ± 0.18	0.41 ± 0.15*†	0.49 ± 0.28*†‡
PR, beats/min²	0.38 ± 0.19	0.33 ± 0.26*	0.39 ± 0.2†	0.28 ± 0.14*†‡
**Range**				
SBP, mm Hg	51.5 ± 14.66	52.1 ± 13.46	64.39 ± 19.38*†	66.2 ± 18.4*†
DBP, mm Hg	49.32 ± 17.28	48.08 ± 15.44	62.53 ± 20.54*†	59.61 ± 21.06*†
MBP, mm Hg	49.26 ± 17.22	48.36 ± 14.48	62.6 ± 19.83*†	59.82 ± 18.72*†
PP, mm Hg	38.8 ± 10.27	39.51 ± 12.2	45.3 ± 12.21*†	54.97 ± 18.42*†‡
PR, beats/min	62.25 ± 32.59	51.42 ± 32.04*	66.64 ± 36.44†	45.27 ± 27.43*†‡
**Peak**				
SBP, mm Hg	26.67 ± 11.43	27.04 ± 9.56	34.23 ± 16.79*†	33.83 ± 11.92*†
DBP, mm Hg	27.8 ± 15.06	26.96 ± 13.4	37.16 ± 18.86*†	35.44 ± 18.04*†
MBP, mm Hg	27.53 ± 14.66	26.83 ± 12.02	36.32 ± 18.24*†	33.27 ± 14.42*†
PP, mm Hg	18.95 ± 6.41	19.02 ± 7.3	22.12 ± 7.66*†	25.67 ± 9.98*†‡
PR, beats/min	41.48 ± 28.74	36.67 ± 30.0*	45.14 ± 33.56†	30.83 ± 25.69*†‡
**Through**				
SBP, mm Hg	24.82 ± 7.24	25.06 ± 6.97	30.16 ± 7.15*†	32.37 ± 10.43*†‡
DBP, mm Hg	21.51 ± 5.44	21.12 ± 5.48	25.37 ± 5.83*†	24.16 ± 6.61*†‡
MBP, mm Hg	21.72 ± 5.81	21.53 ± 5.78	26.28 ± 5.84*†	26.55 ± 7.85*†
PP, mm Hg	19.86 ± 6.31	20.48 ± 7.56	23.18 ± 7.31*†	29.29 ± 12.03*†‡
PR, beats/min	20.77 ± 7.67	14.75 ± 4.52*	21.5 ± 7.0†	14.44 ± 5.82*‡
**Nocturnal Fall**				
SBP, mean (SD)	0.09 ± 0.05	0.08 ± 0.06*	0.12 ± 0.05*†	0.08 ± 0.06*‡
DBP, mean (SD)	0.15 ± 0.07	0.12 ± 0.07*	0.17 ± 0.07*†	0.12 ± 0.07*‡
MBP, mean (SD)	0.12 ± 0.06	0.1 ± 0.06*	0.15 ± 0.06*†	0.1 ± 0.06*‡
PP, mean (SD)	0.01 ± 0.08	0.02 ± 0.08*	0.03 ± 0.08*	0.02 ± 0.1
PR, mean (SD)	0.16 ± 0.08	0.12 ± 0.09*	0.18 ± 0.08*†	0.12 ± 0.08*‡
**Night/Day ratio**				
SBP, mean (SD)	0.91 ± 0.05	0.92 ± 0.06*	0.88 ± 0.05*†	0.92 ± 0.06*‡
DBP, mean (SD)	0.85 ± 0.07	0.88 ± 0.07*	0.83 ± 0.07*†	0.88 ± 0.07*‡
MBP, mean (SD)	0.88 ± 0.06	0.9 ± 0.06*	0.85 ± 0.06*†	0.9 ± 0.06*‡
PP, mean (SD)	0.99 ± 0.08	0.98 ± 0.08*	0.97 ± 0.08*	0.98 ± 0.1
PR, mean (SD)	0.84 ± 0.08	0.88 ± 0.09*	0.82 ± 0.08*†	0.88 ± 0.08*‡
**Morning surge**				
SBP, mm Hg	11.72 ± 10.5	10.49 ± 10.44	17.34 ± 11.43*†	18.19 ± 14.05*†
DBP, mm Hg	10.34 ± 9.09	9.09 ± 9.37*	15.63 ± 10.64*†	14.53 ± 10.77*†
MBP, mm Hg	10.79 ± 9.36	9.31 ± 9.42*	16.28 ± 10.46*†	15.47 ± 11.36*†
PP, mm Hg	1.38 ± 6.0	1.4 ± 6.08	1.71 ± 7.13	3.66 ± 9.38*†‡
PR, mm Hg	9.74 ± 12.08	5.77 ± 10.23*	13.05 ± 12.96*†	8.37 ± 10.07†‡

Values are mean (±SD) or number of subjects (%). Significance for between-clusters differences: *P < 0.05 vs Cluster 1; †P < 0.05 vs Cluster 2; ‡P < 0.05 vs Cluster 3. SBP, systolic blood pressure; DBP, diastolic blood pressure; MBP, Mean blood pressure; PP, pulse pressure; PR, pulse rate.

### 24-Hour ABPM and incident CV events

**Fig 4:**
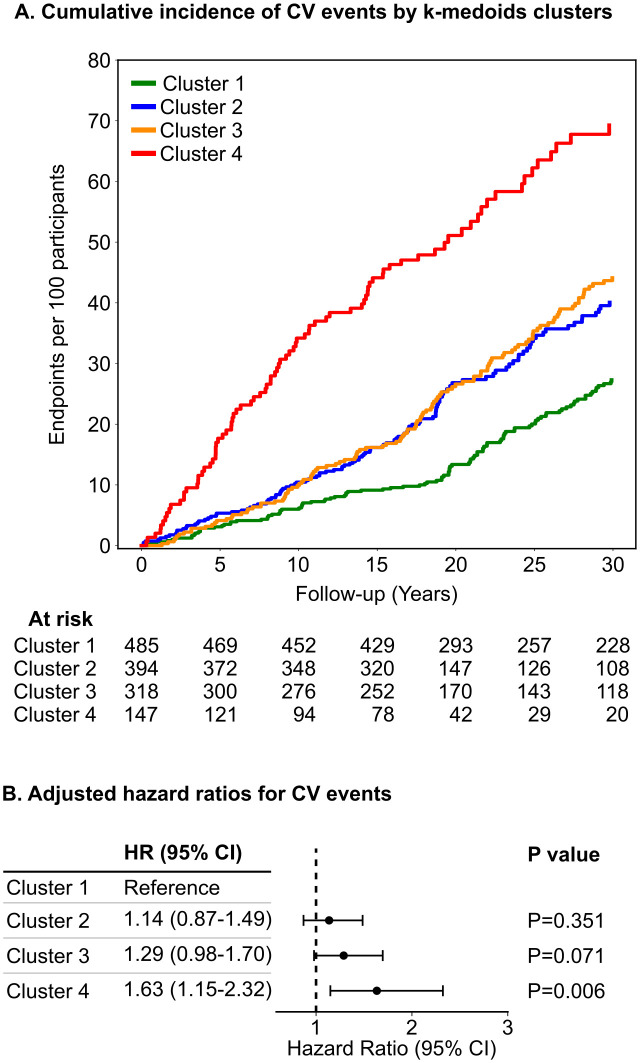
Risk for major CV events by 24-hour ABPM clusters derived by the k-medoids model in FLEMENGHO participants. Panel A shows the incidence of adverse events per cluster. Panel B presents the Cox regression hazard ratios (95% Cl) for CV events per cluster relative to cluster 1. The model was adjusted for: age, sex, body mass index, smoking, drinking, office systolic blood pressure, total cholesterol, diabetes mellitus, history of CV disease and antihypertensive medication intake.

In the training cohort the median follow-up time was 19.4 years (5^th^ to 95^th^ percentile 4.3-37.7 years). During 31092 person-years of follow-up, a total of 470 individuals experienced at least one CV event, reaching an event rate of 15.1 events per 1000 person-years. Fig 4, panel A presents the cumulative incidence of CV events for the 24-hour ABPM clusters. Cluster 1 had the lowest incidence of events, with 122 outcomes and a rate of 9.7 per 1,000 person-years. Clusters 2 and 3 had similar risk, with 127 events (14.8/1,000 person-years) and 132 (17.5/1,000 person-years) respectively. Cluster 4 showed the highest risk of developing adverse CV events, recording 89 events at a rate of 37.6 per 1,000 person-years.

The adjusted HRs showed that participants assigned to cluster 4 had significantly higher risk to develop adverse CV events (Fig 4, panel B). Specifically, after adjusting for traditional CV risk factors at baseline, cluster 4 had a 63% higher risk compared to cluster 1 (reference). Cluster 4 remained significant after adjustment for SCORE2/SCORE2-OP in a subset of 829 FLEMENGHO participants for whom risk scores could be calculated according to current guidelines. Compared with cluster 1 participants in cluster 4 had a 55% higher risk of incident CV events (HR: 1.55; 95% CI: 1.12–2.14; P = 0.008; S3 Table). Notably, only total cholesterol (P = 0.0477) and history of CV disease (P < 0.005) were violating the proportional hazard assumptions. However, the residuals did not show any trend with respect to time (S3 Fig). As such, the observed deviations can be considered small and unlikely meaningful to affect the model’s estimations.

### External validation

**Fig 5:**
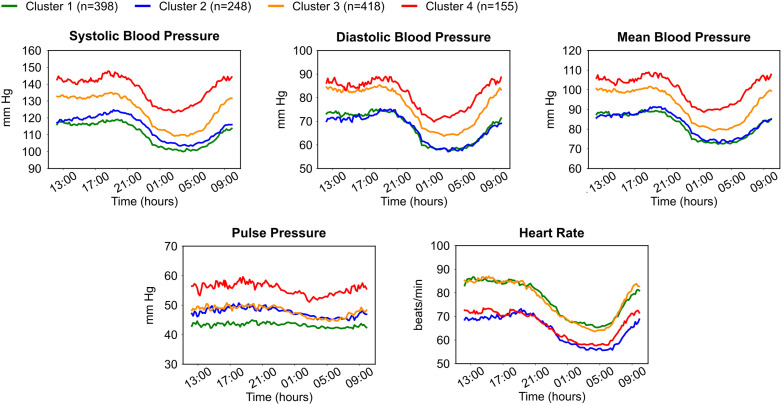
24-hour ABPM trends by k-medoids clusters in EPOGH participants. The trends are calculated as the average of the individual 24-hour ABPM traces assigned to each cluster.

**Fig 6:**
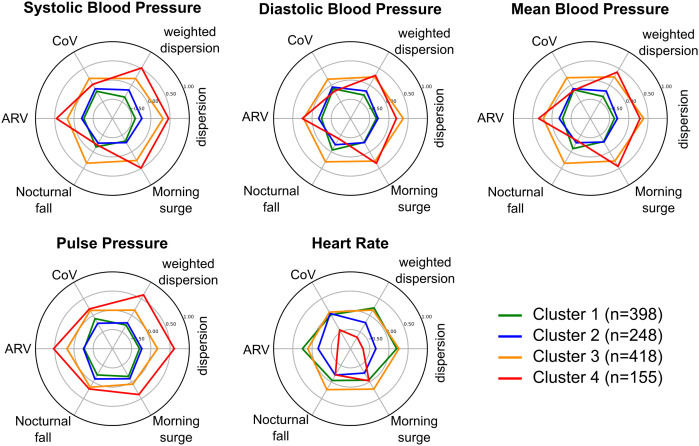
Z-score of Blood pressure variability by k-medoids clusters in EPOGH participants. Positive z-score indicate higher values than the mean value of the EPOGH cohort and negative indicate lower. CoV represents coefficient of variation and ARV average real variability.

To validate the clinical relevance and generalizability of our approach, we applied the trained model to 24-hour ABPM recordings from the EPOGH cohort. Using the medoids obtained from the FLEMENGHO datasets, we assigned EPOGH participants to the nearest medoid. It should be mentioned that the EPOGH cohort showed similar to FLEMENGHO distribution of the distances from the medoids. Both cohorts showed similar overall distributions, although the EPOGH cohort was slightly shifted to the right. Notably, cluster 4, which represented the highest CV risk group, had an almost identical distribution in both cohorts (S4 Fig). The validation cohort included 1219 participants, of whom 650 (53.3%) were women. Compared to the FLEMENGHO cohort, the EPOGH cohort comprised younger individuals (mean age, 39.1 years), with considerably fewer adverse CV events (n = 86). The median follow-up time was 13.03 years (5^th^-95^th^ percentile 4.8-17.1; 13450 person-years). The derived clusters followed the patterns observed in FLEMENGHO, with cluster 4 showing the highest 24-hour SBP and DBP profiles and cluster 1 the lowest (Fig 5). Additionally, clinical characteristics and BP variability indices revealed comparable profiles across clusters (S4 Table, Fig 6). Similarly, the incidence of CV events (S5 Fig) was higher in cluster 4 (27 events; 16.7/1,000 person-years) compared to cluster 1 (15 events; 3.3/1,000 person-years).

## Discussion

In this study we employed a multivariate time series clustering approach to construct 24-hour ABPM profiles. Our analysis revealed 4 clusters with distinct differences in the 24-hour ABPM profiles. Specifically, groups 1 and 2 had similar 24-hour SBP and DBP profiles but they differed significantly in the heart rate profile. On the other hand, group 4 showed the highest 24-hour SBP profile over the whole 24-hour period and the highest 24-hour DBP profile during night-time but one of the lowest heart rate trends. Additionally, clusters 3 and 4 were associated with the highest BPV including dispersion, coefficient of variation and nocturnal fall, diverging from each other mostly in nocturnal fall with cluster 3 reporting higher values. At the same time, survival analysis showed that the participants assigned to cluster 4 had significantly higher risk to develop adverse CV events beyond office SBP and other traditional risk factors covariables that used to calculate conventional risk score.

Over the past decades, the well-demonstrated prognostic importance of 24-hour ABPM has turned ABPM into the recommended method to ascertain hypertension [4,12]. Following the report by Perloff et al [20], several studies in both the general population [21–25] and hypertensive patients [26–28] showed that 24-hour mean SBP and/or DBP are important predictors for CV risk independently from office BP. Our analysis of the time series data through unsupervised ML validated this knowledge. The profiles extracted from the derived clusters showed that cluster 1 had the lowest 24-hour ABPM profiles within the normotensive range and the lowest CV risk, while cluster 4 had the highest 24-hour ABPM with significantly higher CV risk. In summary, cluster 1 represented a normotensive BP profile with a higher HR, though still within the normal range. Cluster 2 displayed also a normotensive BP pattern with a lower HR. Cluster 3 showed elevated BP levels, along with increased BPV and greater nocturnal dipping. Cluster 4 corresponded to a hypertensive profile with high BPV.

Along the prognostic importance of 24-ABPM, BPV has also emerged as an important characteristic to evaluate CV risk. There are several reports presenting a variety of BPV indices that can predict incident CV events independently of 24-hour ABPM [6,8,29]. However, the abundance of BPV indices, combined with the inconclusive relationship between BPV and CV risk [30] hindered the inclusion in guidelines for the management of hypertension so far. Additionally, there are indices such as morning surge for which there is no consensus on how to calculate them, complicating their assessment as important predictors for CV events [11].

Along this line, a computational model analysing time-series 24-hour ABPM recordings might potentially improve and facilitate the clinical interpretation of this data. Indeed, by including the raw ABPM data, a model could capture both the average BP trend and its inherent variability properties. Also, given the high dimensionality of the time series data and the complex interrelations between the available measurements, non-linear machine/deep learning models should be used in this context. Despite the fast growth of artificial intelligence and the introduction of deep learning models in medicine, to our knowledge no research has been conducted on a ML approach that integrates multivariable 24-hour ABPM recordings.

In this study, we have shown that multivariate ML algorithms might complement the current approach of clinical interpretation of 24-hour ABPM recordings. Our findings were consistent with current knowledge and showed that cluster 4 with the highest BP profiles had higher values in several BPV indices, as well as the highest CV risk. As shown in our study, CV outcomes were primarily associated with BP profiles but HR. Although clusters 1 and 2 demonstrated different HR profiles, there was no difference in CV risk between them. Of note, participants in cluster 2 reported higher intake of beta-blockers and other antihypertensive medication which can affect the HR.

Considering also the increasing impact of wearables, [31,32] such as smartwatches, computational intelligent systems could play a crucial role in detecting elevated BP and potentially impede its progression to hypertension. Consequently, this study could be considered as the first step towards integrative profiling and automatic assessment of 24-hour ABPM in population.

It should be highlighted that our computational approach aimed to evaluate the ability of unsupervised ML models to automatically distinguish individuals into distinct risk groups. Towards this direction, the derived clusters fell well within the current BP classification. For example, cluster 1 profiles corresponded to normotensive individuals, clusters 2 and 3 displayed progressively elevated BP profiles, and cluster 4 represented mostly hypertensive individuals with markedly higher 24-hour ABPM values. In this study, we therefore propose a complementary approach to interpreting multivariate 24-hour ABPM recordings, one that facilitates rapid interpretation and visualization of individual 24-hour BP parameters together with BPV, while avoiding the need to calculate those multiple BPV coefficients separately. It also should be noted that major hypertensive guidelines do not provide formal cut-offs for BPV indexes, therefore they are not commonly used in clinical practice. With an ML-guided approach, we are able to integrate all key information derived from 24-hour ABPM time series data.

However, more studies in diverse and multi-ethnic cohorts are warranted to ascertain the clinical importance of the proposed approach. Finally, integrating information regarding the degree of activity or rest of the participants could potentially improve the interpretation of the variability in 24-hour ABPM recordings.

### Limitations

Although the data acquisition was highly standardized, the sampling rate varied among the participants. However, by limiting the missing measurements to 3 hours maximum, we ascertained that the recordings contained sufficient measurements during both day and night hours. Additionally, DTW treated ABPM measurements as equally spaced by index, which could lead to a relative underrepresentation of nighttime segments. However, the acquisition protocol was the same across all participants allowing us to capture trajectory similarities. Furthermore, our analysis showed significant differences in nocturnal dipping in both systolic and diastolic BP suggesting that the trained model captured nighttime BP patterns. Although the proposed model was validated on a multicentre dataset, further and extensive validation would strengthen its applicability in clinical practice. In particular, validating the model in ethnically diverse datasets would provide insightful information regarding the model’s clinical importance. Finally, evaluating the model’s performance using recordings from wearable devices could further expand the range of its applicability.

## Conclusion

In this study unsupervised learning algorithms applied to raw 24-hour ABPM recordings facilitated the integration of 24-hour BP values and BPV into one model, thereby potentially improving the clinical interpretation of this data. The 24-hour ABPM clusters showed distinct BP and BPV profiles and differed independently in CV risk. This model can potentially be incorporated into commercial software solutions for a more complete clinical interpretation of ABPM recordings.

## Supporting information

S1 Text 24-Hour ABPM Database. (DOCX)

S2 Text Clustering Algorithm. (DOCX)

S3 Text Cluster Stability Analysis. (DOCX)

S1 Table Summary table with the formulae used to calculate the blood pressure variability indices. (DOCX)

S2 Table Summary table with hypertension definitions. (DOCX)

S3 Table Adjusted hazard ratios with cluster 1 as reference. The model was adjusted only for SCORE2/SCORE2-OP. A subset of 829 FLEMENGHO participants was used, as SCORE2/SCORE2-OP is only applicable to individuals aged 40 years or older.
(DOCX)

S4 Table Clinical characteristics and 24-hour ABPM of EPOGH participants by k-medoids clusters. (DOCX)

S1 Fig Overview of the computational pipeline. Blue and orange parallelograms illustrate the input data and the output of the processing steps respectively. Green rounded rectangles indicate data processing steps. The flow of the steps is represented by black arrows. ABPM; ambulatory blood pressure monitoring, SBP; systolic blood pressure, DBP; diastolic blood pressure; HR; heart rate, DTW; dynamic time warping.
(DOCX)

S2 Fig Consensus matrix for three different number of clusters. Left panel: Consensus matrix for 3 clusters. Middle panel: Consensus matrix for 4 clusters. Right panel: Consensus matrix for 5 clusters. Each row and column represents a training example and the colour of each cell indicates the co-clustering probability. The closer to 1, the more often the two training examples are clustered in the same group. The 4-cluster solution (middle panel) provided the clearest, most stable pattern with well-defined blocks, uniform intensities and good separation between clusters.
(DOCX)

S3 Fig Schoenfeld residuals of the cofounders used to adjust the Cox regression model. Statistically only total cholesterol and history of CV disease revealed violation of the proportional hazard assumption. However, there is not apparent trend with respect to the time. As such, the Cox regression model is assumed to remain robust.
(DOCX)

S4 Fig Distributions of the distances from the derived medoids for FLEMENGHO (blue) and EPOGH (orange) cohorts per cluster. Both cohorts showed similar overall distributions, although the EPOGH cohort was slightly shifted to the right. Notably, cluster 4, which represented the highest CV risk group, had an almost identical distribution in both cohorts.
(DOCX)

S5 Fig Risk for major CV events by 24-h ABPM clusters derived by the k-medoids model in EPOGH participants. (DOCX)
