# Supplementary material for: Machine learning–driven integration of 24-hour ambulatory blood pressure and its variability
Source: PLOS Digit Health. 2026 Jul 16;5(7):e0001499. doi: 10.1371/journal.pdig.0001499 (PMC13374967; doi:10.1371/journal.pdig.0001499)
Supplement: S1 Text — (DOCX) [file pdig.0001499.s001.docx]

**24-Hour ABPM Database**

All 24-hour ABPM time series were stored in text files containing in addition participant identifier, and recording date. Time was recorded in seconds, with the first measurement for the time series analysis defined as the one taken immediately after midnight. For each measurement, heart rate, systolic and diastolic blood pressure, and the sequential order of measurement (starting at 1) were recorded. An additional column in our dataset provided the weight of each measurement, calculated from the time interval in minutes between recordings. Measurement quality was coded as 0 (poor quality: >3 consecutive hours missing) or 1 (good quality). A total of 1,512 recordings were excluded due to poor quality: 1,376 due to completely missing night-time measurements, 2 due to more than 3 consecutive hours missing during night-time, 131 due to more than 3 consecutive hours missing during day-time, and 3 due to complete recording failure. No editing of individual measurements was performed.
