# Supplementary material for: Machine learning–driven integration of 24-hour ambulatory blood pressure and its variability
Source: PLOS Digit Health. 2026 Jul 16;5(7):e0001499. doi: 10.1371/journal.pdig.0001499 (PMC13374967; doi:10.1371/journal.pdig.0001499)
Supplement: S2 Text — (DOCX) [file pdig.0001499.s002.docx]

**Clustering Algorithm**

Dynamic time warping (DTW) is a distance metric with a main objective to find the temporal alignment between two time series curves (either univariate or multivariate) that minimizes the Euclidean distance. Formally, this is described by the following optimization function:

$$DTW\left( x, y \right)=\min_{\pi} \sqrt{\sum_{(i, j)\in\pi} {d(x_{i},y_{i})}^{2}}$$

where $x=(x_{0}, \ldots, x_{n-1})$ and $y=(y_{0}, \ldots, y_{m-1})$ are the two time series sequences and $\pi=\left[ \pi_{0}, \ldots, \pi_{K} \right]$ is a the alignment path that satisfies the following properties:

1. $\pi_{\kappa}=\left( i_{k}, j_{k} \right)$ with $0\leq i_{k}\leq n$ and $0\leq j_{k}\leq m$
2. $\pi_{0}=\left( 0, 0 \right)$ and $\pi_{K}=\left( n-1, m-1 \right)$
3. For all k>0, $\pi_{\kappa}$ is related to $\pi_{\kappa-1}$ as follows:
   1. $i_{k-1}\leq i_{k}\leq i_{k-1}+1$
   2. $j_{k-1}\leq j_{k}\leq j_{k-1}+1$

We opted for DTW as (i) it can handle data of different length, (ii) it is suitable for multivariate data analysis and (iii) it can capture differences in both the shape and the values of the time series sequences. Therefore, DTW does not require temporally aligned sequences and as such data imputation is not required.

Finally, k-medoids clustering algorithm was selected due to its robustness to noise and outliers. K-medoids forms the cluster centres (i.e. medoids) using actual observations rather than average values making it more suitable for heterogeneous clinical data.
