# Supplementary material for: Machine learning–driven integration of 24-hour ambulatory blood pressure and its variability
Source: PLOS Digit Health. 2026 Jul 16;5(7):e0001499. doi: 10.1371/journal.pdig.0001499 (PMC13374967; doi:10.1371/journal.pdig.0001499)
Supplement: S3 Text — (DOCX) [file pdig.0001499.s003.docx]

**Cluster Stability Analysis**

To evaluate the clustering stability, we repeatedly fitted the clustering model on random subsets of the data. For each run (in total we used 30 runs), a fraction of samples was selected without replacement, clustered and used to update a consensus matrix. The latter records how often airs of observation are grouped together. Cluster labels from different runs are aligned using the Hungarian algorithm to ensure consistent label matching before computing confusion matrices across overlapping samples. The averaged confusion matrix is then used to derive both an overall stability score and per-cluster stability values. We iterated this process for 3, 4 and 5 clusters.
