# Supplementary material for: Machine learning–driven integration of 24-hour ambulatory blood pressure and its variability
Source: PLOS Digit Health. 2026 Jul 16;5(7):e0001499. doi: 10.1371/journal.pdig.0001499 (PMC13374967; doi:10.1371/journal.pdig.0001499)
Supplement: S1 Table — (DOCX) [file pdig.0001499.s004.docx]

**S1 Table**: Summary table with the formulae used to calculate the blood pressure variability indices.

| **Blood pressure variability** | **Formula** |
| --- | --- |
| Dispersion | $SD=\sqrt{\frac{1}{N-1}\sum_{i=1}^{N} {(BP - \bar{BP})}^{2}}$ |
| Weighted dispersion | ${SD}_{w}= \frac{{SD}_{day}\times n_{day}+ {SD}_{night}\times n_{night}}{n_{day} + n_{night}}$ |
| Average real variability | $ARV= \frac{1}{N-1} \times\sum_{i=1}^{N-1} \left\vert{BP}_{i+1} - {BP}_{i} \right\vert$ |
| Time rate | $\mathrm{TR}= \frac{1}{N-1} \times\sum_{i=1}^{N-1} \frac{\left\vert{BP}_{i+1} - {BP}_{i} \right\vert}{t_{i+1}- t_{i}}$ |
| Range | $Range=\max\left( BP \right)-\min(BP)$ |
| Peak | $Peak=\max\left( BP \right)- \bar{BP}$ |
| Through | $Through= \bar{BP}-\min(BP)$ |
| Nocturnal Fall | $NF= \frac{\bar{{BP}_{Day}}-\bar{{BP}_{Night}}}{\bar{{BP}_{Day}}}$ |
| Night/Day Ratio | $ND= \frac{\bar{{BP}_{Night}}}{\bar{{BP}_{Day}}}$ |
| Morning Surge | $MS= {BP}_{morning}-{BP}_{Low sleep}$ |
| Day and night are defined according to long fixed clock-time periods. Day corresponds to a time between 7 am and 10 pm. Night corresponds to a time between 10 pm and 7 am. BP, blood pressure; $\bar{{BP}_{i}}$, average value of blood pressure. | |
