# Supplementary material for: Machine learning–driven integration of 24-hour ambulatory blood pressure and its variability
Source: PLOS Digit Health. 2026 Jul 16;5(7):e0001499. doi: 10.1371/journal.pdig.0001499 (PMC13374967; doi:10.1371/journal.pdig.0001499)
Supplement: S2 Table — (DOCX) [file pdig.0001499.s005.docx]

**S2 Table**: Summary table with hypertension definitions.

|  | **Definition** |
| --- | --- |
| In-office hypertension | Office SBP ≥ 140 and/or  office DBP ≥ 90 and/or  antihypertensive medication intake |
| White-coat hypertension | Office SBP ≥ 140 and/or  office DBP ≥ 90 and  no antihypertensive medication intake and  average 24-hour ABPM (SBP/DBP) < 130/80 and  average Daytime ABPM (SBP/DBP) < 135/85 and  average Nighttime ABPM (SBP/DBP) < 120/70 |
| Masked hypertension | Office SBP < 140 and  office DBP < 90 and  no antihypertensive medication intake and  average 24-hour ABPM (SBP/DBP) ≥ 130/80 and/or  average Daytime ABPM (SBP/DBP) ≥135/85 and/or  average Nighttime ABPM (SBP/DBP) ≥120/70 |
| Sustained hypertension | In-office hypertension (see definition above) and  average 24-hour ABPM (SBP/DBP) ≥ 130/80 and/or  average Daytime ABPM (SBP/DBP) ≥135/85 and/or  average Nighttime ABPM (SBP/DBP) ≥120/70 |
| 24-hour ABPM, daytime and nighttime represented time-weighted average values. SBP, Systolic blood pressure; DBP, Diastolic blood pressure; ABPM, Ambulatory blood pressure monitoring | |
