# Supplementary material for: Machine learning–driven integration of 24-hour ambulatory blood pressure and its variability
Source: PLOS Digit Health. 2026 Jul 16;5(7):e0001499. doi: 10.1371/journal.pdig.0001499 (PMC13374967; doi:10.1371/journal.pdig.0001499)
Supplement: S3 Table — The model was adjusted only for SCORE2/SCORE2-OP. A subset of 829 FLEMENGHO participants was used, as SCORE2/SCORE2-OP is only applicable to individuals aged 40 years or older. (DOCX) [file pdig.0001499.s006.docx]

**S3 Table**: Adjusted hazard ratios with cluster 1 as reference. The model was adjusted only for SCORE2/SCORE2-OP. A subset of 829 FLEMENGHO participants was used, as SCORE2/SCORE2-OP is only applicable to individuals aged 40 years or older.

|  | **Hazard Ratio** | **95% CI** | **P value** |
| --- | --- | --- | --- |
| **Cluster 1 (reference)** | - | - | - |
| **Cluster 2** | 1.30 | 1.00-1.69 | 0.050 |
| **Cluster 3** | 1.24 | 0.95-1.62 | 0.116 |
| **Cluster 4** | 1.55 | 1.12-2.14 | 0.008 |
| **SCORE2/SCORE2-OP** | 1.11 | 1.09-1.12 | <0.001 |
