# Supplementary material for: Machine learning–driven integration of 24-hour ambulatory blood pressure and its variability
Source: PLOS Digit Health. 2026 Jul 16;5(7):e0001499. doi: 10.1371/journal.pdig.0001499 (PMC13374967; doi:10.1371/journal.pdig.0001499)
Supplement: S4 Table — (DOCX) [file pdig.0001499.s007.docx]

**S4 Table**: Clinical characteristics and 24-hour ABPM of EPOGH participants by k-medoids clusters.

|  | Cluster 1  (n=398) | Cluster 2  (n=248) | Cluster 3  (n=418) | Cluster 4  (n=155) |
| --- | --- | --- | --- | --- |
| **Anthropometrics** |  |  |  |  |
| Females, n(%) | 289 (72.61) | 105 (42.34)* | 191 (45.69)* | 65 (41.94)* |
| Age, mean (SD), years | 34.28 (13.18) | 37.2 (16.02)* | 40.53 (13.47)*† | 50.58 (11.9)*†‡ |
| Body mass index, mean (SD), kg/m² | 23.85 (4.53) | 24.97 (4.87)* | 26.31 (4.6)*† | 27.47 (4.09)*†‡ |
| Systolic pressure, mean (SD), mm Hg | 114.72 (13.24) | 120.57 (14.09)* | 130.51 (15.46)*† | 146.95 (19.57)*†‡ |
| Diastolic pressure, mean (SD), mm Hg | 73.98 (9.31) | 74.47 (10.25) | 83.07 (11.48)*† | 89.34 (12.72)*†‡ |
| Heart rate, mean (SD), beats/min | 75.97 (9.01) | 67.62 (7.8)* | 75.92 (9.45)† | 69.1 (9.39)*‡ |
| **Questionnaire data** |  |  |  |  |
| Drinking alcohol, n(%) | 116 (29.15) | 72 (29.03) | 159 (38.04)*† | 71 (45.81)*† |
| Current smoking, n(%) | 91 (22.86) | 54 (21.77) | 141 (33.73)*† | 29 (18.71)‡ |
| History of CV diseases, n(%) | 38 (9.55) | 41 (16.53)* | 61 (14.59)* | 47 (30.32)*†‡ |
| History of DM, n(%) | 9 (2.26) | 9 (3.63) | 24 (5.74)* | 7 (4.52) |
| **Medication** |  |  |  |  |
| Diuretics Medication, n(%) | 8 (2.01) | 14 (5.65)* | 30 (7.18)* | 28 (18.06)*†‡ |
| Beta Blockers, n(%) | 7 (1.76) | 28 (11.29)* | 23 (5.5)*† | 31 (20.0)*†‡ |
| Angiotensin Medication, n(%) | 11 (2.76) | 27 (10.89)* | 44 (10.53)* | 50 (32.26)*†‡ |
| Calcium Channel Blocker, n(%) | 5 (1.26) | 8 (3.23) | 18 (4.31)* | 26 (16.77)*†‡ |
| Anti-hypertensive Medication, n(%) | 21 (5.28) | 44 (17.74)* | 74 (17.7)* | 70 (45.16)*†‡ |
| **Hypertension** |  |  |  |  |
| Masked hypertension, n(%) | 20 (5.03) | 22 (8.87) | 104 (24.88)*† | 39 (25.16)*† |
| White coat hypertension, n(%) | 14 (3.52) | 11 (4.44) | 26 (6.22) | 4 (2.58) |
| Sustained hypertension, n(%) | 22 (5.53) | 45 (18.15)* | 145 (34.69)*† | 109 (70.32)*†‡ |
| **Biochemical data** |  |  |  |  |
| Blood glucose, mean (SD), mmol/L | 4.77 (1.14) | 4.87 (0.8) | 5.07 (1.14)*† | 5.16 (1.05)*† |
| Total cholesterol, mean (SD), mmol/L | 4.79 (1.07) | 4.87 (1.12) | 5.28 (1.18)*† | 5.34 (1.04)*† |
| HDL cholesterol, mean (SD), mmol/L | 1.36 (0.42) | 1.3 (0.37) | 1.26 (0.38)* | 1.25 (0.42)* |
| LDL cholesterol, mean (SD), mmol/L | 2.97 (0.96) | 3.05 (1.01) | 3.41 (1.04)*† | 3.5 (1.03)*† |
| Serum creatinine, mean (SD), μmol/L | 76.21 (14.08) | 82.52 (15.26)* | 81.94 (15.09)* | 83.24 (15.27)* |
| **Ambulatory blood pressure** |  |  |  |  |
| **24-hour Systolic blood pressure** |  |  |  |  |
| Mean 24-h ABPM, mean (SD), mm Hg | 111.15 (5.72) | 114.74 (7.44)* | 125.08 (7.21)*† | 137.77 (9.93)*†‡ |
| Mean day-time, mean (SD), mm Hg | 115.65 (6.03) | 119.02 (7.76)* | 131.56 (7.56)*† | 143.11 (10.53)*†‡ |
| Mean night-time, mean (SD), mm Hg | 103.33 (7.2) | 107.27 (9.0)* | 113.72 (9.21)*† | 128.39 (12.06)*†‡ |
| Day-night difference, mean (SD), mm Hg | 12.32 (6.33) | 11.75 (7.39) | 17.84 (8.18)*† | 14.72 (10.4)*†‡ |
| **24-hour Diastolic blood pressure** |  |  |  |  |
| Mean 24-h ABPM, mean (SD), mm Hg | 67.84 (4.5) | 67.26 (5.67) | 76.91 (5.97)*† | 82.04 (7.74)*†‡ |
| Mean day-time, mean (SD), mm Hg | 72.16 (4.87) | 71.08 (5.86)* | 82.49 (6.1)*† | 86.3 (8.04)*†‡ |
| Mean night-time, mean (SD), mm Hg | 60.34 (5.67) | 60.59 (7.03) | 67.14 (7.56)*† | 74.56 (9.04)*†‡ |
| Day-night difference, mean (SD), mm Hg | 11.81 (5.3) | 10.49 (5.82)* | 15.35 (6.17)*† | 11.75 (6.88)‡ |
| **24-hour Mean blood pressure** |  |  |  |  |
| Mean 24-h ABPM, mean (SD), mm Hg | 82.54 (4.29) | 83.29 (5.44) | 93.05 (5.67)*† | 101.28 (7.94)*†‡ |
| Mean day-time, mean (SD), mm Hg | 86.81 (4.69) | 87.09 (5.71) | 98.84 (5.83)*† | 105.82 (8.43)*†‡ |
| Mean night-time, mean (SD), mm Hg | 75.12 (5.54) | 76.65 (6.86)* | 82.91 (7.53)*† | 93.3 (9.43)*†‡ |
| Day-night difference, mean (SD), mm Hg | 11.69 (5.36) | 10.44 (5.98)* | 15.93 (6.57)*† | 12.52 (7.93)†‡ |
| **24-hour Pulse pressure** |  |  |  |  |
| Mean 24-h ABPM, mean (SD), mm Hg | 43.31 (5.26) | 47.48 (6.93)* | 48.18 (6.45)* | 55.72 (7.66)*†‡ |
| Mean day-time, mean (SD), mm Hg | 43.5 (5.57) | 47.94 (7.21)* | 49.07 (6.97)*† | 56.8 (8.32)*†‡ |
| Mean night-time, mean (SD), mm Hg | 42.98 (5.52) | 46.68 (7.17)* | 46.59 (6.69)* | 53.83 (7.94)*†‡ |
| Day-night difference, mean (SD), mm Hg | 0.51 (3.7) | 1.26 (4.0)* | 2.49 (4.93)*† | 2.97 (6.04)*† |
| **24-hour Heart rate** |  |  |  |  |
| Mean 24-h ABPM, mean (SD), mm Hg | 77.64 (5.81) | 64.91 (4.73)* | 77.4 (7.03)† | 66.96 (7.06)*†‡ |
| Mean day-time, mean (SD), mm Hg | 82.84 (6.72) | 68.71 (5.27)* | 83.05 (7.63)† | 70.91 (7.59)*†‡ |
| Mean night-time, mean (SD), mm Hg | 68.59 (7.22) | 58.27 (5.86)* | 67.52 (8.21)*† | 59.99 (7.43)*†‡ |
| Day-night difference, mean (SD), mm Hg | 14.25 (7.78) | 10.44 (5.81)* | 15.53 (7.26)*† | 10.92 (5.48)*‡ |
| **Blood pressure variability** |  |  |  |  |
| **Dispersion** |  |  |  |  |
| SBP, mean (SD), mm Hg | 12.16 (3.42) | 13.1 (3.89)* | 15.39 (4.53)*† | 16.2 (4.44)*† |
| DBP, mean (SD), mm Hg | 11.01 (2.71) | 11.27 (2.97) | 13.35 (3.8)*† | 12.78 (3.5)*† |
| MBP, mean (SD), mm Hg | 10.83 (2.61) | 11.17 (2.93) | 13.33 (3.57)*† | 13.27 (3.26)*† |
| PP, mean (SD), mm Hg | 8.22 (3.01) | 8.7 (3.34) | 10.24 (4.25)*† | 11.79 (4.56)*†‡ |
| PR, mean (SD), beats/min | 13.82 (3.79) | 11.71 (3.5)* | 13.83 (3.63)† | 10.16 (2.95)*†‡ |
| **weighted Dispersion** |  |  |  |  |
| SBP, mean (SD), mm Hg | 10.46 (3.23) | 11.45 (3.64)* | 12.72 (4.16)*† | 14.16 (3.82)*†‡ |
| DBP, mean (SD), mm Hg | 9.32 (2.61) | 9.85 (2.87)* | 11.01 (3.62)*† | 11.17 (3.33)*† |
| MBP, mean (SD), mm Hg | 9.16 (2.48) | 9.74 (2.75)* | 10.91 (3.3)*† | 11.57 (2.84)*†‡ |
| PP, mean (SD), mm Hg | 7.91 (2.84) | 8.38 (3.11) | 9.72 (3.92)*† | 11.23 (4.22)*†‡ |
| PR, mean (SD), beats/min | 11.6 (3.2) | 10.25 (3.21)* | 11.3 (3.05)† | 8.51 (2.63)*†‡ |
| **Coefficient of variation** |  |  |  |  |
| SBP, mean (SD) | 10.85 (3.1) | 11.29 (3.28) | 12.18 (3.57)*† | 11.66 (3.1)* |
| DBP, mean (SD) | 16.07 (4.14) | 16.59 (4.43) | 17.25 (5.25)* | 15.52 (4.49)†‡ |
| MBP, mean (SD) | 12.99 (3.21) | 13.26 (3.44) | 14.19 (3.93)*† | 13.01 (3.16)‡ |
| PP, mean (SD) | 19.02 (6.91) | 18.31 (6.9) | 21.12 (8.1)*† | 21.08 (7.71)*† |
| PR, mean (SD) | 17.52 (4.55) | 17.76 (5.06) | 17.65 (4.63) | 15.03 (4.35)*†‡ |
| **Average real variability** |  |  |  |  |
| SBP, mean (SD), mm Hg | 8.77 (2.74) | 9.05 (3.12) | 10.23 (3.57)*† | 11.11 (3.33)*†‡ |
| DBP, mean (SD), mm Hg | 7.7 (2.31) | 8.04 (2.64) | 9.09 (3.48)*† | 9.11 (3.22)*† |
| MBP, mean (SD), mm Hg | 7.52 (2.06) | 7.8 (2.36) | 8.8 (2.87)*† | 9.14 (2.36)*† |
| PP, mean (SD), mm Hg | 7.93 (2.75) | 8.18 (2.98) | 9.68 (3.87)*† | 10.68 (4.29)*†‡ |
| PR, mean (SD), beats/min | 8.68 (2.43) | 7.73 (2.64)* | 8.35 (2.41)† | 6.28 (1.86)*†‡ |
| **Time rate** |  |  |  |  |
| SBP, mean (SD), mm Hg/min | 0.52 (0.23) | 0.52 (0.23) | 0.62 (0.31)*† | 0.66 (0.27)*† |
| DBP, mean (SD), mm Hg/min | 0.46 (0.19) | 0.48 (0.22) | 0.56 (0.31)*† | 0.55 (0.25)*† |
| MBP, mean (SD), mm Hg/min | 0.44 (0.17) | 0.46 (0.19) | 0.54 (0.27)*† | 0.54 (0.2)*† |
| PP, mean (SD), mm Hg/min | 0.49 (0.23) | 0.5 (0.23) | 0.61 (0.32)*† | 0.67 (0.35)*† |
| PR, mean (SD), beats/min² | 0.52 (0.21) | 0.46 (0.19)* | 0.51 (0.19)† | 0.37 (0.13)*†‡ |
| **Range** |  |  |  |  |
| SBP, mean (SD), mm Hg | 58.3 (21.04) | 62.23 (21.14)* | 71.83 (25.08)*† | 76.11 (23.63)*† |
| DBP, mean (SD), mm Hg | 52.19 (17.8) | 52.39 (17.88) | 62.11 (22.08)*† | 62.65 (21.75)*† |
| MBP, mean (SD), mm Hg | 52.08 (17.65) | 52.47 (17.09) | 61.78 (19.92)*† | 63.12 (17.14)*† |
| PP, mean (SD), mm Hg | 44.64 (18.85) | 46.65 (20.42) | 54.48 (24.67)*† | 62.37 (26.27)*†‡ |
| PR, mean (SD), beats/min | 64.01 (20.57) | 56.46 (21.42)* | 65.26 (21.9)† | 49.05 (18.55)*†‡ |
| **Peak** |  |  |  |  |
| SBP, mean (SD), mm Hg | 31.82 (17.54) | 33.62 (17.11) | 38.47 (19.13)*† | 41.0 (16.2)*† |
| DBP, mean (SD), mm Hg | 28.79 (16.23) | 28.83 (15.73) | 33.92 (19.28)*† | 34.16 (17.7)*† |
| MBP, mean (SD), mm Hg | 28.93 (15.54) | 28.67 (14.4) | 33.46 (16.76)*† | 34.62 (13.69)*† |
| PP, mean (SD), mm Hg | 24.98 (14.9) | 25.81 (14.97) | 30.94 (18.62)*† | 35.17 (18.15)*†‡ |
| PR, mean (SD), beats/min | 39.91 (17.32) | 37.38 (18.31) | 40.3 (18.66)† | 31.33 (15.16)*†‡ |
| **Through** |  |  |  |  |
| SBP, mean (SD), mm Hg | 26.48 (6.83) | 28.61 (7.38)* | 33.35 (9.32)*† | 35.11 (10.78)*† |
| DBP, mean (SD), mm Hg | 23.4 (4.59) | 23.56 (4.75) | 28.19 (5.98)*† | 28.48 (7.23)*† |
| MBP, mean (SD), mm Hg | 23.15 (5.0) | 23.8 (5.31) | 28.32 (6.43)*† | 28.51 (6.81)*† |
| PP, mean (SD), mm Hg | 19.65 (6.36) | 20.84 (7.63)* | 23.54 (8.7)*† | 27.2 (10.54)*†‡ |
| PR, mean (SD), beats/min | 24.1 (7.75) | 19.08 (6.22)* | 24.96 (8.03)† | 17.72 (6.19)*†‡ |
| **Nocturnal fall** |  |  |  |  |
| SBP, mean (SD) | 0.1 (0.05) | 0.1 (0.06) | 0.13 (0.06)*† | 0.1 (0.07)‡ |
| DBP, mean (SD) | 0.16 (0.07) | 0.14 (0.08)* | 0.18 (0.07)*† | 0.13 (0.07)*‡ |
| MBP, mean (SD) | 0.13 (0.06) | 0.12 (0.07)* | 0.16 (0.06)*† | 0.11 (0.07)*‡ |
| PP, mean (SD) | 0.01 (0.08) | 0.03 (0.08)* | 0.05 (0.09)*† | 0.05 (0.09)*† |
| PR, mean (SD) | 0.17 (0.08) | 0.15 (0.08)* | 0.18 (0.08)*† | 0.15 (0.07)*‡ |
| **Night/Day ratio** |  |  |  |  |
| SBP, mean (SD) | 0.9 (0.05) | 0.9 (0.06) | 0.87 (0.06)*† | 0.9 (0.07)‡ |
| DBP, mean (SD) | 0.84 (0.07) | 0.86 (0.08)* | 0.82 (0.07)*† | 0.87 (0.07)*‡ |
| MBP, mean (SD) | 0.87 (0.06) | 0.88 (0.07)* | 0.84 (0.06)*† | 0.89 (0.07)*‡ |
| PP, mean (SD) | 0.99 (0.08) | 0.97 (0.08)* | 0.95 (0.09)*† | 0.95 (0.09)*† |
| PR, mean (SD) | 0.83 (0.08) | 0.85 (0.08)* | 0.82 (0.08)*† | 0.85 (0.07)*‡ |
| **Morning surge** |  |  |  |  |
| SBP, mean (SD), mm Hg | 13.32 (10.46) | 12.97 (11.74) | 20.43 (13.18)*† | 24.05 (16.21)*†‡ |
| DBP, mean (SD), mm Hg | 11.62 (9.23) | 10.62 (9.56) | 16.95 (11.11)*† | 18.51 (11.45)*† |
| MBP, mean (SD), mm Hg | 11.86 (9.08) | 11.01 (9.83) | 17.8 (11.54)*† | 20.45 (12.43)*†‡ |
| PP, mean (SD), mm Hg | 1.7 (5.86) | 2.35 (6.62) | 3.48 (7.54)* | 5.54 (10.35)*†‡ |
| PR, mean (SD), beats/min | 8.68 (13.51) | 5.49 (11.31)* | 12.03 (12.38)*† | 9.27 (10.22)†‡ |
| Values are mean (SD) or number of subjects (%). Significance for between-phenogroups differences: *P<0.05 vs Cluster 1; †P<0.05 vs Cluster 2; ‡P<0.05 vs Cluster 3. HDL, high density lipoprotein; LDL, low density lipoprotein; SBP, systolic blood pressure; DBP, diastolic blood pressure; MBP, Mean blood pressure; PP, pulse pressure; PR, pulse rate. | | | | |
