# Supplementary material for: Machine learning–driven integration of 24-hour ambulatory blood pressure and its variability
Source: PLOS Digit Health. 2026 Jul 16;5(7):e0001499. doi: 10.1371/journal.pdig.0001499 (PMC13374967; doi:10.1371/journal.pdig.0001499)

**S1 Figure**: Overview of the computational pipeline. Blue and orange parallelograms illustrate the input data and the output of the processing steps respectively. Green rounded rectangles indicate data processing steps. The flow of the steps is represented by black arrows. ABPM; ambulatory blood pressure monitoring, SBP; systolic blood pressure, DBP; diastolic blood pressure; HR; heart rate, DTW; dynamic time warping.


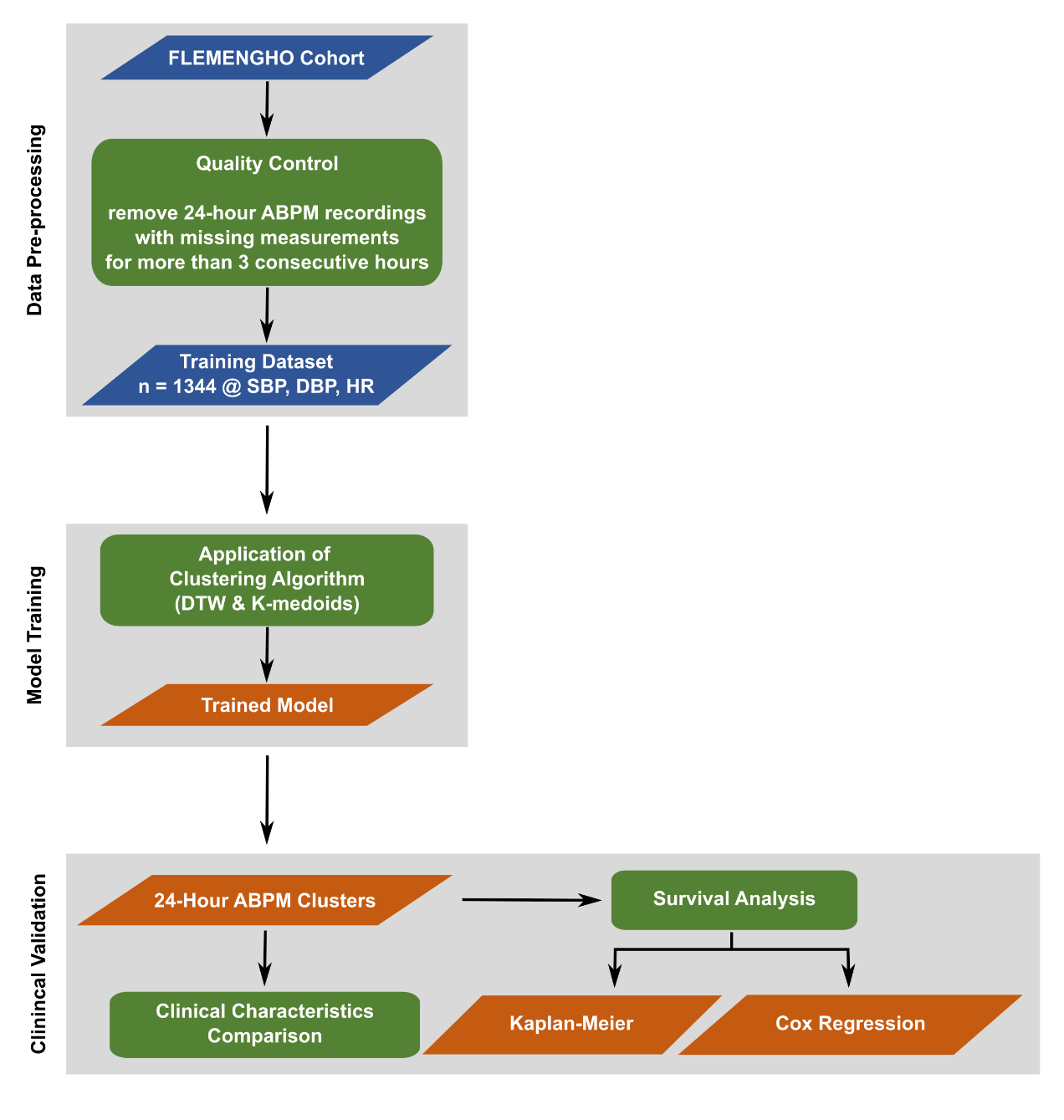

Supplement: S1 Fig — Blue and orange parallelograms illustrate the input data and the output of the processing steps respectively. Green rounded rectangles indicate data processing steps. The flow of the steps is represented by black arrows. ABPM; ambulatory blood pressure monitoring, SBP; systolic blood pressure, DBP; diastolic blood pressure; HR; heart rate, DTW; dynamic time warping. (DOCX) [file pdig.0001499.s008.docx]
