# Supplementary material for: Machine learning–driven integration of 24-hour ambulatory blood pressure and its variability
Source: PLOS Digit Health. 2026 Jul 16;5(7):e0001499. doi: 10.1371/journal.pdig.0001499 (PMC13374967; doi:10.1371/journal.pdig.0001499)
Supplement: S2 Fig — Left panel: Consensus matrix for 3 clusters. Middle panel: Consensus matrix for 4 clusters. Right panel: Consensus matrix for 5 clusters. Each row and column represents a training example and the colour of each cell indicates the co-clustering probability. The closer to 1, the more often the two training examples are clustered in the same group. The 4-cluster solution (middle panel) provided the clearest, most stable pattern with well-defined blocks, uniform intensities and good separation between clusters. (DOCX) [file pdig.0001499.s009.docx]

**S2 Figure**: Consensus matrix for three different number of clusters. Left panel: Consensus matrix for 3 clusters. Middle panel: Consensus matrix for 4 clusters. Right panel: Consensus matrix for 5 clusters. Each row and column represents a training example and the colour of each cell indicates the co-clustering probability. The closer to 1, the more often the two training examples are clustered in the same group. The 4-cluster solution (middle panel) provided the clearest, most stable pattern with well-defined blocks, uniform intensities and good separation between clusters.

**
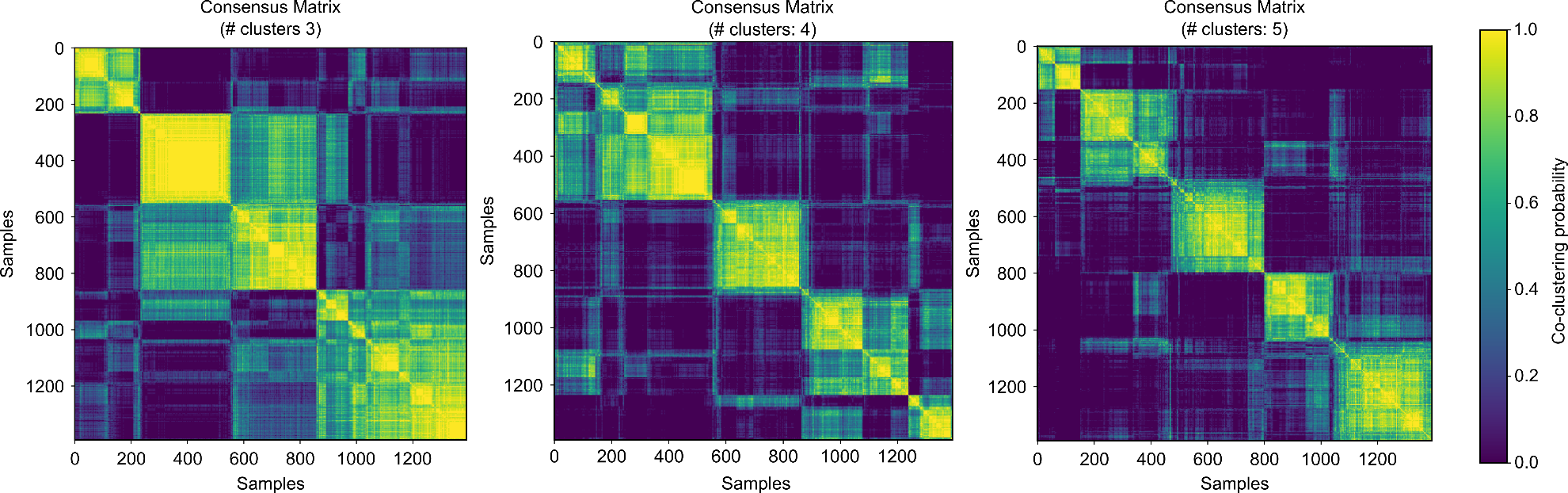
**
