# Supplementary material for: Machine learning–driven integration of 24-hour ambulatory blood pressure and its variability
Source: PLOS Digit Health. 2026 Jul 16;5(7):e0001499. doi: 10.1371/journal.pdig.0001499 (PMC13374967; doi:10.1371/journal.pdig.0001499)

**S3 Figure:** Schoenfeld residuals of the cofounders used to adjust the Cox regression model. Statistically only total cholesterol and history of CV disease revealed violation of the proportional hazard assumption. However, there is not apparent trend with respect to the time. As such, the Cox regression model is assumed to remain robust.


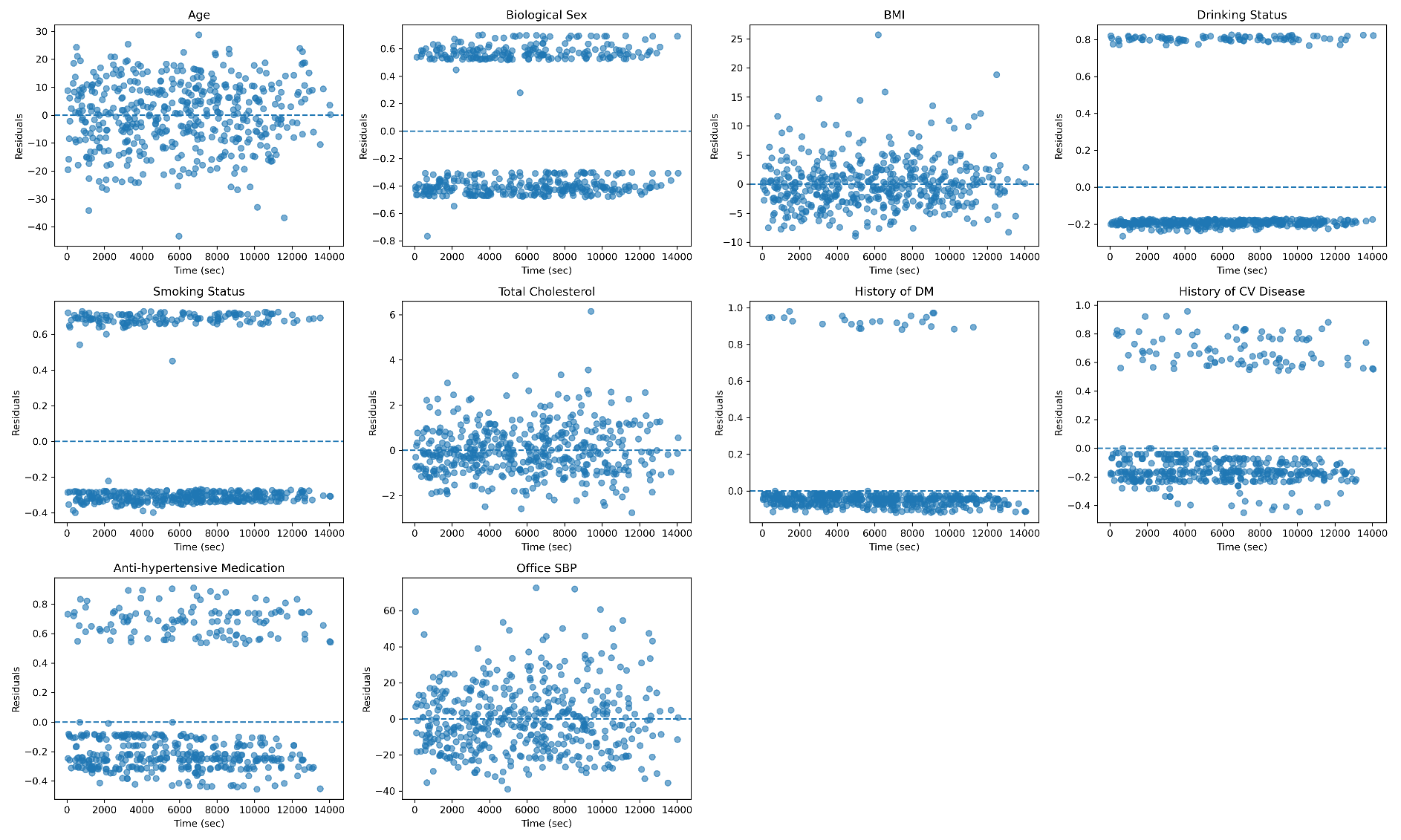

Supplement: S3 Fig — Statistically only total cholesterol and history of CV disease revealed violation of the proportional hazard assumption. However, there is not apparent trend with respect to the time. As such, the Cox regression model is assumed to remain robust. (DOCX) [file pdig.0001499.s010.docx]
