# Supplementary material for: Machine learning–driven integration of 24-hour ambulatory blood pressure and its variability
Source: PLOS Digit Health. 2026 Jul 16;5(7):e0001499. doi: 10.1371/journal.pdig.0001499 (PMC13374967; doi:10.1371/journal.pdig.0001499)
Supplement: S4 Fig — Both cohorts showed similar overall distributions, although the EPOGH cohort was slightly shifted to the right. Notably, cluster 4, which represented the highest CV risk group, had an almost identical distribution in both cohorts. (DOCX) [file pdig.0001499.s011.docx]

**S4 Figure:** Distributions of the distances from the derived medoids for FLEMENGHO (blue) and EPOGH (orange) cohorts per cluster. Both cohorts showed similar overall distributions, although the EPOGH cohort was slightly shifted to the right. Notably, cluster 4, which represented the highest CV risk group, had an almost identical distribution in both cohorts.

**
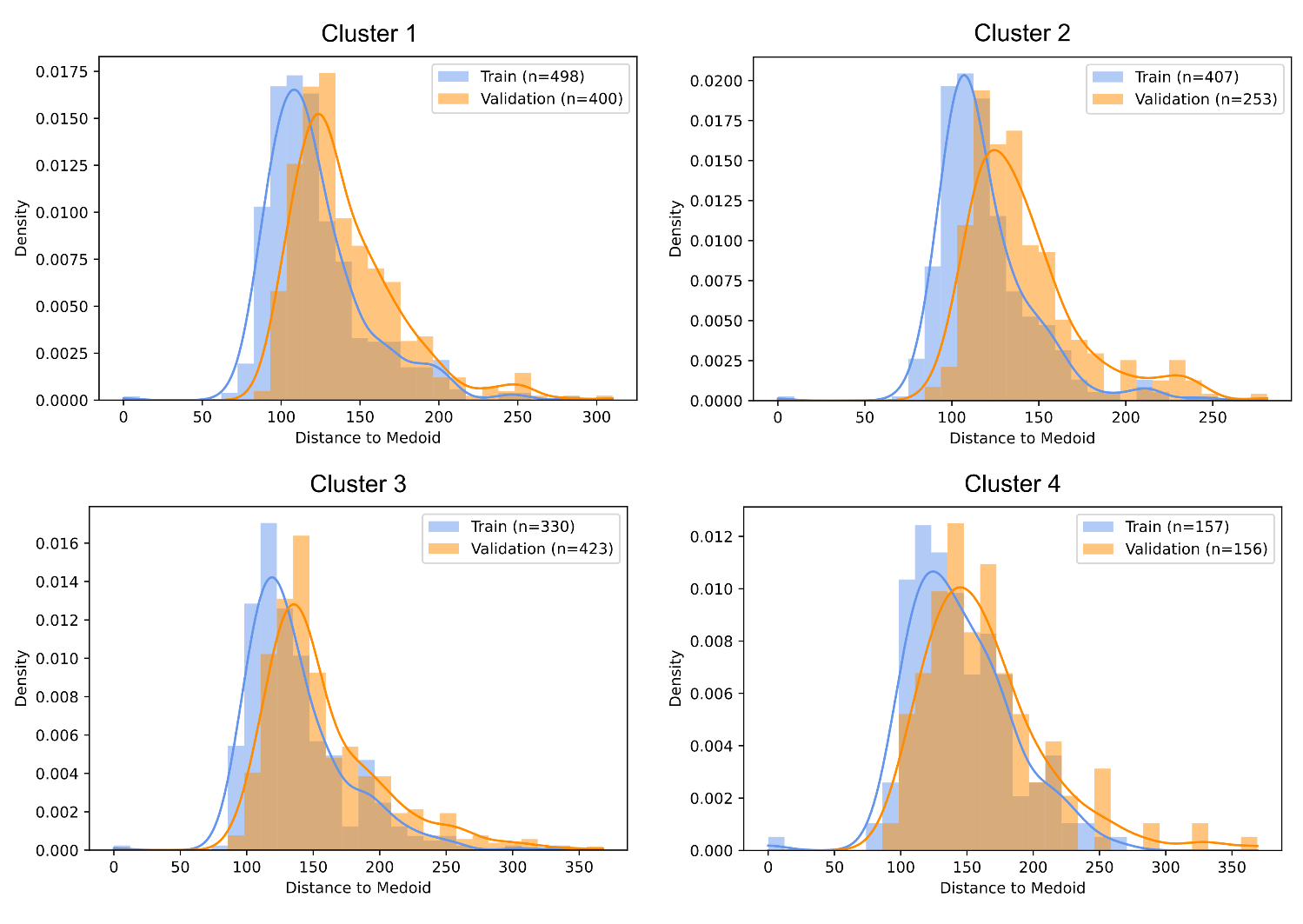
**
