# Supplementary material for: Machine learning–driven integration of 24-hour ambulatory blood pressure and its variability
Source: PLOS Digit Health. 2026 Jul 16;5(7):e0001499. doi: 10.1371/journal.pdig.0001499 (PMC13374967; doi:10.1371/journal.pdig.0001499)

**S5 Figure**: Risk for major CV events by 24-h ABPM clusters derived by the k-medoids model in EPOGH participants.


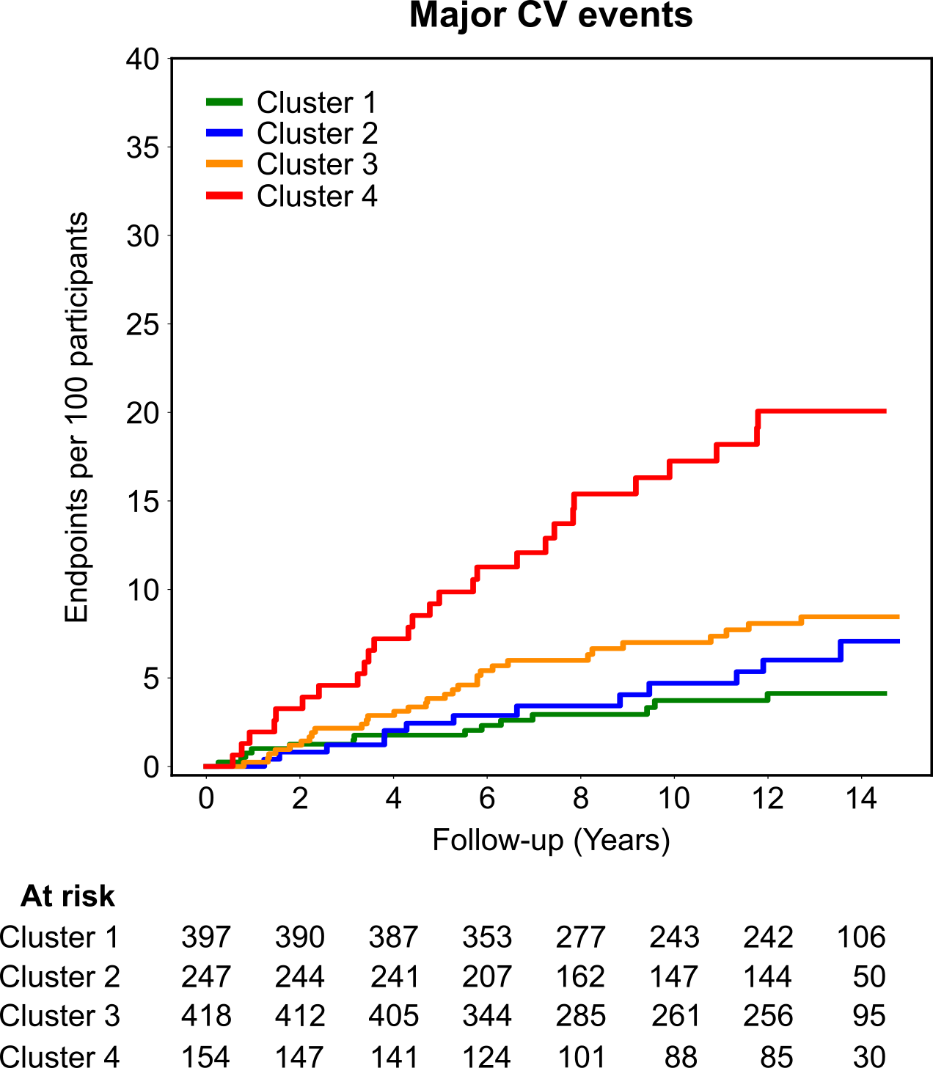

Supplement: S5 Fig — (DOCX) [file pdig.0001499.s012.docx]
