# Supplementary material for: Machine learning–driven integration of 24-hour ambulatory blood pressure and its variability
Source: PLOS Digit Health. 2026 Jul 16;5(7):e0001499. doi: 10.1371/journal.pdig.0001499 (PMC13374967; doi:10.1371/journal.pdig.0001499)
Supplement: Attachment — Submitted filename: Reviewer comments_14022026.docx [file pdig.0001499.s014.docx]

**Reviewer #1:**

**Major comments:**

**1. Cluster 4 clearly has the highest 24‑h SBP/DBP and the highest BPV, and also the highest event rate. However, the Cox models adjust for office SBP and standard risk factors, but not for mean 24‑h SBP (or day/night SBP or simple dipping pattern). The Discussion and Author Summary imply added information beyond existing ABPM interpretation and beyond “24‑hour ambulatory SBP”. To support this key claim, please: (i) add models including mean 24‑h SBP (and/or conventional ABPM categories) and then cluster membership, and report whether clusters still predict events; (ii) Ideally, compare model performance (C‑statistics, IDI/NRI or likelihood ratio tests) between: (a) office risk factors, (b) office + mean 24‑h SBP (±dipping/BPV), and (c) office + ABPM summaries + cluster membership; and (iii) clarify the wording throughout if the clustering does *not* provide independent information beyond mean 24‑h SBP.**

We would like to thank the reviewer for this comment as it provides us the opportunity to discuss the issue of collinearities in a regression model. Our model and resulting clusters were built directly on the 24-h ABPM time series recordings. By definition, these raw recordings already encode information about average 24-h, daytime, and nighttime values. Consequently, including these summary measures as additional covariates in the Cox regression model would introduce correlated predictors, compromising model reliability. This issue has been thoroughly investigated. Back to 1968, Gordon (Gordon RA. Issues in Multiple Regression. AJS. 1968;73(5):592-616. DOI: 10.1086/224533) distinguished between two forms of collinearity: *redundancy* (highly correlated independent variables that may even prevent model convergence) and *repetitiveness* (a larger number of correlated predictors). In both cases, correlated variables can lead to instability and misleading results.

We also wish to emphasize that the present study does not aim to establish new thresholds or diagnostic criteria for hypertension. Rather, we propose an alternative and user-friendly way using a novel computational approach to interpret raw 24-h ABPM recordings by bypassing the often tedious calculation of multiple summary parameters and especially BP variability indices—many of which lack standardized definitions. To clarify this objective, we have added the following paragraph in the Discussion section.

*“**It should be highlighted that our computational approach aimed to evaluate the ability of unsupervised ML models to automatically distinguish individuals into distinct risk groups. Towards this direction, the derived clusters fell well within the current BP classification. For example, cluster 1 profiles corresponded to normotensive individuals, clusters 2 and 3 displayed progressively elevated BP profiles, and cluster 4 represented mostly hypertensive individuals with markedly higher 24-h ABPM values. In this study, we therefore propose a complementary approach to interpreting multivariate 24-h ABPM recordings, one that facilitates rapid interpretation and visualization of individual 24-h BP parameters together with BPV, while avoiding the need to calculate those multiple BPV coefficients separately. It also should be noted that major hypertensive guidelines do not provide formal cut-offs for BPV indexes, therefore they are not commonly used in clinical practice. With an ML-guided approach, we are able to integrate all key information derived from 24h-ABPM time series data.”* (lines 323-336)*.*

Although including highly-correlated variables in any regression model including the Cox regression as mentioned above might lead to questionable results due to model instability, we investigated the impact of the derived phenogroups by fitting multiple Cox regression with several 24-h ABPM parameters as requested. Table 1 below shows the hazard ratios (with cluster 1 as reference) after adjusting for age sex, BMI, dirking and smoking habits, total cholesterol, history of DM and CV diseases, anti-hypertensive treatment, office SBP, average day-time ambulatory systolic BP and average real variability of 24-h ambulatory systolic BP. Notably, cluster 5 remained significant showing a 51% increase in the risk of developing adverse CV events when compared to cluster 1.

**Table 1**: Adjusted hazard ratios with cluster 1 as reference. The model was adjusted for age, sex, BMI, drinking, smoking, total cholesterol, history of DM, history of CV diseases, antihypertensive treatment, office SBP, the average day-time ambulatory SBP and average real variability of 24-h ambulatory SBP.

|  | **Hazard Ratio** | **95% CI** | **P value** |
| --- | --- | --- | --- |
| **Cluster 1 (reference)** | - | - | - |
| **Cluster 2** | 1.13 | 0.86-1.48 | 0.385 |
| **Cluster 3** | 1.23 | 0.91-1.65 | 0.180 |
| **Cluster 4** | 1.51 | 1.00-2.27 | 0.048 |
| **Office SBP** | 1.00 | 0.99-1.00 | 0.325 |
| **Average day-time SBP** | 1.00 | 0.99-1.01 | 0.643 |
| **Average Real Variability (SBP)** | 1.02 | 0.98-1.06 | 0.460 |

When we included several highly correlated variables in the same model, namely the 24-h ABPM clusters, office SBP, mean 24-h SBP, and BPV AVR, none of them reached statistical significance. This suggests that the model is unstable, likely due to multicollinearity (Table 2). In contrast, when we included in the model only the cluster variables (Table 3) or only the mean 24-h SBP (Table 4), each was statistically significant. These findings support our interpretation that cluster labels and mean 24-h SBP are colinear variables. As we previously stated, because the ABPM variables capture overlapping information, including them in the same model weakens their individual effects and obscures their association with the outcome.

**Table 2**: Adjusted hazard ratios with cluster 1 as reference. The model was adjusted for age, sex, BMI, drinking, smoking, total cholesterol, history of DM, history of CV diseases, antihypertensive treatment, office SBP, the average 24-h ambulatory SBP and average real variability of 24-h ambulatory SBP.

|  | **Hazard Ratio** | **95% CI** | **P value** |
| --- | --- | --- | --- |
| **Cluster 1 (reference)** | - | - | - |
| **Cluster 2** | 1.1167 | 0.853-1.463 | 0.420 |
| **Cluster 3** | 1.194 | 0.893-1.596 | 0.232 |
| **Cluster 4** | 1.395 | 0.928-2.098 | 0.110 |
| **Office SBP** | 0.995 | 0.988-1.002 | 0.193 |
| **Average 24-h SBP** | 1.008 | 0.996-1.020 | 0.197 |
| **Average Real Variability (SBP)** | 1.010 | 0.969-1.051 | 0.655 |

**Table 3**: Adjusted hazard ratios with cluster 1 as reference. The model was adjusted for age, sex, BMI, drinking, smoking, total cholesterol, history of DM, history of CV diseases, antihypertensive treatment, office SBP.

|  | **Hazard Ratio** | **95% CI** | **P value** |
| --- | --- | --- | --- |
| **Cluster 1 (reference)** | - | - | - |
| **Cluster 2** | 1.136 | 0.869-1.486 | 0.351 |
| **Cluster 3** | 1.288 | 0.979-1.695 | 0.071 |
| **Cluster 4** | 1.634 | 1.149-2.323 | 0.006 |
| **Office SBP** | 0.997 | 0.991-1.004 | 0.401 |

**Table 4**: Adjusted hazard ratios without including the clustering labels. The model was adjusted for age, sex, BMI, drinking, smoking, total cholesterol, history of DM, history of CV diseases, antihypertensive treatment, office SBP and average of 24-h SBP.

|  | **Hazard Ratio** | **95% CI** | **P value** |
| --- | --- | --- | --- |
| **Office SBP** | 0.996 | 0.989-1.003 | 0.299 |
| **Average 24-h SBP** | 1.013 | 1.004-1.023 | 0.005 |

Please, also refer to our reply to the question 4 (iii sub question).

**2. You state that raw ABPM recordings were used “without any signal processing” and that k‑medoids with dynamic time warping was applied to SBP, DBP and HR time series. Please clarify:**

i. **How irregular sampling (20‑min daytime, 45‑min night) and occasional missing readings were handled in the DTW distance (regular resampling? inclusion of timestamps? simple sequence index?).**

As mentioned in the supplemental data “24-h ABPM database”, line 18, *“No editing of the individual measurements was performed”.* We opted to use directly the raw time series recordings as DTW allows the comparison of time series data of uneven length.

ii. **Whether SBP, DBP and HR were scaled/standardized before computing multivariate DTW.**

We clarified in the main manuscript that we used the raw time series recordings with no scaling or standardization as we wanted to capture information related to both dynamic fluctuations and the BP level. Specifically, in the “Data processing and analysis” section (lines 156-159) we added: *“We used the raw time series recordings without performing any scaling (or standardization) to capture both temporal characteristics (dynamic fluctuations) of the 24-h ABPM and the BP levels”*.

iii. **The exact multivariate DTW implementation (e.g., independent DTW per channel averaged vs joint multivariate DTW) and package used.**

We added the requested information in the “Data processing and analysis” section, line 159. In particular we clearly describe that *“DTW was calculated jointly for all variables using “Tslearn” library”*.

iv. **Provide at least a brief assessment of cluster stability (e.g., bootstrapping or Jaccard similarity) and a sensitivity analysis to different numbers of clusters (k=3,5) beyond the single hybrid CVI curve on page 30.**

To evaluate the clustering stability, we repeatedly fitted the clustering model on random subsets of the data. For each run (in total we used 30 runs), a fraction of samples was selected without replacement, clustered and used to update a consensus matrix. The latter records how often airs of observation are grouped together. Cluster labels from different runs are aligned using the Hungarian algorithm to ensure consistent label matching before computing confusion matrices across overlapping samples. The averaged confusion matrix is then used to derive both an overall stability score and per-cluster stability values. We iterated this process for 3, 4 and 5 clusters.

For all three potential clusters we observed similar overall stability, which reached 0.73 for 3 clusters, 0.69 for 4 and 0.70 for 5 clusters. Additionally, Figure below shows the consensus matrices for 3, 4 and 5 clusters. Each row and column represents a training example and the colour of each cell indicates the co-clustering probability, values close to 1 mean that two examples were always placed in the same cluster whereas values closer to 0 indicate they were rarely grouped together. High-intensity blocks with sharp boundaries separated by darker regions would suggest well-separated and reproducible clusters.

The three consensus matrix figures illustrate the effect of different cluster member on data structure. The 3-cluster solution (left panel) shows strong blocks and high internal consensus, but one block is disproportionately large and contains internal texture, indicating under-clustering and hidden structure. The 4-cluster solution (middle panel) provided the clearest, most stable pattern with well-defined blocks, uniform intensities and separation between clusters. On the contrary, 5-cluster solution (right panel) showed thinner blocks with less sharp boundaries and lower intensities, which could be an indication of over-clustering where naturally cohesive groups are unnecessarily split. It should be highlighted that samples that seem to change clusters between the runs is an indication of overlapping clusters, which is very commonly observed in physiological data. From these results we can safely conclude that 4 clusters was indeed the optimal number of clusters as was defined initially by hybrid CVI curve. This information included in Supplemental data of the revised manuscript.

**3. In EPOGH, it is stated that the “trained model” was applied and four clusters were again observed. Please specify clearly whether:**

i. **The FLEMENGHO medoids were fixed and each EPOGH subject was assigned to the nearest existing medoid, or clustering was re‑run de novo in EPOGH.**

We would like to thank the reviewer for this insightful comment. In the revised manuscript we clarified that the FLEMENGHO medoids were used to assign each EPOGH participant to the nearest medoid. Specifically in “External Validation” paragraph (lines 251-253) we wrote:

*“Using the medoids obtained from the FLEMENGHO datasets, we assigned EPOGH participants to the nearest medoid.”*

ii. **If medoids were reused, describe any distributional differences between cohorts and whether distances to medoids were similar. If clustering was repeated, then it is not a strict external validation of the *same* unsupervised structure and this should be acknowledged.**

As requested, we compared the distances from the medoids for each cluster separately by comparing their respective distributions (see Figure below). *Both cohorts showed similar overall distributions, although the EPOGH cohort was slightly shifted to the right. Notably, cluster 4, which represented the highest CV risk group, had an almost identical distribution in both cohorts* (lines 253-256).

**4. Clusters 1 and 2 have very similar SBP/DBP profiles but differ mainly in HR; clusters 3 and 4 show higher BP and BPV. It would help readers if you:**

i. **Provide clearer clinical labels/interpretation (e.g., “normotensive/high HR”, “intermediate BP/high dipping”, “hypertensive/high variability”).**

As requested, in the revised Discussion we provided a clearer clinical labels (lines 288-292). Particularly, we included the following explanation:

*“In summary, cluster 1 represented a normotensive BP profile with a higher HR, though still within the normal range. Cluster 2 displayed also a normotensive BP pattern with a lower HR. Cluster 3 showed elevated BP levels, along with increased BPV and greater nocturnal dipping. Cluster 4 corresponded to a hypertensive profile with high BPV”.*

ii. **Comment more explicitly on why HR‑driven differentiation between clusters 1 and 2 does not appear to translate into clearly different risk, and whether HR adds anything once BP level is accounted for.**

We commented on the effect of heart rate on CV risk in the “Discussion” section (lines 314-317). Specifically we included the following text:

*“As shown in our study, CV outcomes were primarily associated with BP profiles but HR. Although clusters 1 and 2 demonstrated different HR profiles, there was no difference in CV risk between them. Of note, participants in cluster 2 reported higher intake of beta blockers and other antihypertensive medication which can affect the HR”.*

iii. **Consider a model where cluster membership is replaced by continuous mean 24‑h BP and HR (±BPV indices) to show what additional information the clustering itself contributes.**

To quantify the additional information that the clustering may provide we trained 3 separate Cox regression models. For all 3 models the c-index was compared to assess the predictive performance. Table below summarises the 3 Cox regression model, listing the parameters used for adjustment and the calculated c-index. Additional to the parameters listed below, traditional CV risk factors including age, sex, BMI, total cholesterol, drinking and smoking habits, history of diabetes mellitus and CV disease and the office systolic BP were used

| **# Model** | **Parameters used for adjustment** | **C-index** |
| --- | --- | --- |
| Model 1 | Mean of 24-h SBP  Mean of 24-h HR | 0.8176 |
| Model 2 | Mean of 24-h SBP  Mean of 24-h HR  SBP dispersion  SBP ARV | 0.8181 |
| Model 3 | Cluster labels | 0.8176 |

All 3 model resulted in comparable c-indexes indicating similar predictive performance. Of note, when we included the mean of 24-h DBP in the Cox regression models the 24-h SBP lost its significance. This could already be an indication of the linear models’ instability when colinear parameters are included.

Overall, these findings reinforce the point made in the Discussion section that the proposed approach may serve as a complementary tool to automate the integration of 24-h ABPM levels and their variability.

**5. The current Data Availability statement (“data will be shared on reasonable request”) does not meet PLOS’ requirement for open data in a public repository.**

To improve the reliability of our study we provided the scripts used to produce out study’s results. The scripts are available at: <https://github.com/HCVE/24h_abpm_clustering>. Please, note that we must comply to the European GDPR regulations (<https://eur-lex.europa.eu/eli/reg/2016/679/oj> ). Thus, we can provide any access to individual data only after request that should be approved by the local Ethical Commission. Therefore, providing the personal data such as age, sex, bmi, etc online would be a serious privacy violation according to the current GDPR regulations.

**Minor comments:**

**1. Use one term consistently for groups (“clusters” vs “phenogroups”). Currently both are used somewhat interchangeably. Standardize “24‑hour” vs “24-h” across abstract, text and figures.**

The terms “clusters” and “phenogroups” were used to differentiate the results from our computational methodology and the results obtained from the clinical evaluation (clinical characteristics/outcome analysis). However, we recognise that this might confuse readers and therefore we replaces the word “phenogroups” with the “clusters”. Similarly, we standardised the term “24-hour” by retaining the full term and replacing “24-h” where necessary.

**2. You describe comparing continuous variables with the Z distribution and proportions with χ². It may be clearer to state that you used linear/logistic regression or ANOVA/χ² tests as appropriate. Specify how proportional hazards assumptions were checked in the Cox models.**

In the revised version, we provided an clearer explanation of how z- and $\chi^{2}$-distributions were used in our statistical analysis. Specifically, in the “Statistical Analysis” section (lines 170-172) we mentioned that: *“We statistically compared the means of the continuous variables’ distributions using z-test, while to assess differences in the proportions of the continuous variables the* $\chi^{2}$ *statistical test was used”*. As such we would like to clarify that no regression model was used to statistically compare continuous distributions.

The proportional hazard assumptions were evaluated using Schoenfeld residuals. Out of all the variables used to adjust Cox regression model only total cholesterol (P=0.0477) and history of CV disease (P<0.005) emerged as significant. However, when we visually observe the residuals we did not identify any apparent trend with respect to time. Therefore, we can support the argument that no violation of the proportional hazard assumption was observed.

We included the results of this analysis in the revised manuscript, in the “24-h ABPM and incident CV events” section, lines 244-248. Specifically, we wrote that *“Notably, only total cholesterol (P=0.0477) and history of CV disease (P<0.005) were violating the proportional hazard assumptions. However, the residuals did not show any trend with respect to time (Supplemental Figure S3). As such, the observed deviations can be considered small and unlikely meaningful to affect the model’s estimations”*.

**3. Radar plots in Figures 3 and 6 are visually appealing but dense; consider adding a short explanation in the legends of what a positive vs negative Z‑score means relative to the overall cohort and perhaps including one or two key BPV indices in a simpler bar plot in the main text. Please ensure all abbreviations used in Tables 1–3 are defined in the footnotes at first appearance (e.g., “ARV”, “CoV”).**

Following the reviewer’s suggestion we included in the legends of Figure 3 and Figure 6 an explanation of the z-score. Specifically in Figure’s 3 legend we added *“Positive z-scores indicate higher values than the mean value of the FLEMENGHO cohort, while negative z-scores indicate lower values.”* Similarly in Figure’s 6 legend we added *“Positive z-scores indicate higher values than the mean value of the EPOGH cohort, while negative z-scores indicate lower values.”* Furthermore, we ascertained that all abbreviations used in Tables 1-3 are defined in the footnotes.

Finally, introducing additional bar plots would duplicate information already shown and increase the length and visual complexity of the main text without substantially improving interpretability. For these reasons, we have opted to retain the radar-plot format as the primary representation.

**4. The Limitations section could more clearly state that the cohorts are European, with limited ethnic diversity, and that generalizability to other populations, and to contemporary ABPM/wearable devices, remains to be tested.**

In the revised section on limitations, we specified that validation in ethnically diverse datasets is necessary and validation in wearable devices would be beneficial. Specifically we added (lines 347-350): *“**In particular, validating the model in ethnically diverse datasets would provide insightful information regarding the model’s clinical importance.* *Finally, evaluating the model’s performance using recordings from wearable devices could further expand the range of its applicability. ”*.

**Reviewer #2:**

**Please write about the unsupervised ML algorithm you used to cluster datasets. why you have chosen the k-medoids clustering algorithm. Have you compared your choice of algorithm with other algorithms in the unsupervised ML domain? What is the speciality of the K-medoids clustering algorithm?**

We addressed the rationale regarding the selection of the clustering algorithm in the supplemental data (lines 35-37). Specifically, we specified: *“Finally, k-medoids clustering algorithm was selected due to its robustness to noise and outliers. K-medoids forms the cluster centres (i.e. medoids) using actual observations rather than average values making it more suitable for heterogeneous clinical data”*.

In addition, throughout our study we compared k-medoids clustering to other clustering algorithms including k-means and hierarchical clustering, the latter both using DTW and feature extraction. However, using other approaches we were not able to identify a clear separation between clusters regarding values of mean 24-h ABPM, BPV or other clinical characteristics.

**Reviewer #3:**

**Thank you for the opportunity to review this manuscript. The topic—leveraging machine learning to integrate 24-hour ABPM and BP variability—is relevant and aligns with the scope of digital health research. However, several major issues limit the scientific contribution and prevent the manuscript from meeting publication standards in its current form.**

**1. Limited novelty and incremental insight. The unsupervised clustering approach (DTW + k-medoids) applies established methods without demonstrating clear methodological innovation. The resulting four phenogroups largely recapitulate known BP categories (normotensive → hypertensive), as reflected in the ABPM profiles and cluster characteristics (Tables 1–2; Figures 1–2) . The study does not clearly show what new insights are gained beyond conventional ABPM summaries or standard BPV metrics.**

The aim of this study was not to provide new definitions for hypertension or suggest new BPV metrics. On the contrary we aimed to assess if a computational model could integrate both 24-h ABPM values and BPV into a single model. Throughout the discussion section we address the fact that the derived cluster fall within the current definitions of normotensive and hypertensive patterns and that this approach can be considered only as a complementary method of assessing 24-h ABPM. For instance, in lines 323-332, we mention:

*“It should be highlighted that our computational approach aimed to evaluate the ability of unsupervised ML models to automatically distinguish individuals into distinct risk groups. Towards this direction, the derived clusters fell well within the current BP classification. For example, cluster 1 profiles corresponded to normotensive individuals, clusters 2 and 3 displayed progressively elevated BP profiles, and cluster 4 represented hypertensive individuals with markedly higher 24-h ABPM values.* *In this study, we therefore propose a complementary approach to interpreting multivariate 24-h ABPM recordings, one that facilitates rapid interpretation and visualization of individual 24-h BP parameters together with BPV coefficients, while avoiding the need to calculate them separately”.*

This highlights the fact that time series ML could facilitate the integration of multiple metrics (24-h ABPM level and BPV) into a single model making it more appealing for wearable devices and automatic assessment. *It also should be noted that major hypertensive guidelines* ***do not provide formal cut-offs for BPV indexes****, therefore they are not commonly used in clinical practice. Importantly, the contribution of this work is methodological rather than incremental, introducing an ML-guided approach that enables comprehensive integration of 24-h ABPM time-series information* (lines 332-336).

2. **Statistical analysis requires substantial strengthening. Key analytical concerns include: Absence of cluster stability assessments or sensitivity analyses; No evaluation of model calibration, discrimination, or predictive improvement (e.g., C-index, NRI);Limited adjustment for important confounders; Unclear testing of proportional hazards assumptions; Insufficient external validation, with primarily descriptive comparisons (Figure 5–6). Overall, the evidence supporting the clusters’ independent prognostic value remains incomplete**.

We improved our statistical analysis and we strengthened our methodological choices by providing more details regarding our choice on the clustering algorithm, the cluster stability and any violation on the proportional hazard assumptions. Overall, cluster stability analysis (lines 186-189 in main text; please, also refer to comment 2 of Reviewer 1) showed that indeed 4 was the optimal number of clusters for our dataset. Additionally, Schoenfeld residuals revealed no serious violations of the proportional hazards assumptions (lines 244-248 in main text).

**3. Data availability does not meet PLOS requirements. The statement that data “will be shared upon reasonable request” does not comply with PLOS’s open data policy. Raw ABPM time series, cluster assignments, and analysis code must be publicly accessible.**

Please, refer to our reply to comment 5 of Reviewer 1.

**4. Interpretation and clinical implications need clearer articulation. Although the manuscript proposes integrative interpretation of ABPM + BPV, the clinical meaning of each cluster remains insufficiently differentiated. Differences between clusters largely reflect BP level rather than novel temporal or variability phenotypes (Figures 2–3) . More explicit justification for clinical utility is needed.**

We specified the clinical interpretation of our results by firstly providing a more clinical-oriented description of the clusters and by commenting on the effect of HR in the outcome results. In particular, in lines 288-292 we included: *“In summary, cluster 1 represented a normotensive BP profile with a higher HR, though still within the normal range. Cluster 2 displayed also a normotensive BP pattern with a lower HR. Cluster 3 showed elevated BP levels, along with increased BPV and greater nocturnal dipping. Cluster 4 corresponded to a hypertensive profile with high BPV”*.

Additionally, in line 314-317 we specified that: “*As shown in our study, CV outcomes were primarily associated with BP profiles but HR. Although clusters 1 and 2 demonstrated different HR profiles, there was no difference in CV risk between them. Of note, participants in cluster 2 reported higher intake of beta blockers and other anti-hypertensive medication which can affect the HR.”*

Finally, we would like to highlight that this approach does not aim to provide additional parameter to detect hypertension or to provide a new definition. This was already mentioned in the “Discussion” section, where we clearly state that: *“In this study, we therefore propose a complementary approach to interpreting multivariate 24-h ABPM recordings, one that facilitates rapid interpretation and visualization of individual 24-h BP parameters together with BPV coefficients, while avoiding the need to calculate them separately”*.

**5. Presentation and methodological clarity. The manuscript is generally well written, but several methodological choices—such as handling of heterogeneous sampling intervals, rationale for DTW parameters, and pre-processing decisions—require clearer explanation. While the work addresses an important problem, substantial revisions are necessary to enhance methodological robustness, demonstrate true incremental value, and comply with data-sharing requirements. I encourage the authors to strengthen the analytical framework, expand validation, and more clearly define the clinical significance of the proposed phenogroups.**

Following the recommendation of the reviewer we strengthen our methodological choices by including more information regarding the clustering algorithm (lines 156-159), providing information on the cluster stability (lines 186-189) and proportional hazard assumptions (lines 244-248). We also provided a clearer clinical interpretation of the results (lines 252-256 & 314-317 & 323-336).
