# Supplementary material for: Machine learning–driven integration of 24-hour ambulatory blood pressure and its variability
Source: PLOS Digit Health. 2026 Jul 16;5(7):e0001499. doi: 10.1371/journal.pdig.0001499 (PMC13374967; doi:10.1371/journal.pdig.0001499)
Supplement: Attachment — Submitted filename: Reviewers_response_30042026_R3.docx [file pdig.0001499.s015.docx]

Reply to Reviewers:

**Reviewer 1**

1. **Comment 1: The current approach (“shared on reasonable request” and ethics approval needed) is unlikely to meet PLOS requirements as written. PLOS Digital Health requires that data needed to replicate findings be publicly available at publication, and when legal/ethical constraints apply, the statement must specify where the data reside and the exact access mechanism.**

We acknowledge that the data availability statement does not fully meet the PLOS Digital Health requirements for publicly available datasets. However, as mentioned in our previous response the utilised datasets fall under EU GDPR regulations which prohibits public release. As such, access to the data can be granted through written request to the corresponding author. Currently the data are stored in KU Leuven’s digital repository with access limited to the Principal Investigator (and corresponding author Prof. Tatiana Kuznetsova). We added to the Data availability statement to better describe the process of requesting access to the data.

*“The data underlying this article are stored in a digital repository of KU Leuven accessed by the Principal Investigators. The datasets can be shared on a written request to the corresponding author, with clear description of the objectives and the way the data will be used.”*

2. **Comment 2: The added analyses (as described in the response) suggest strong collinearity between cluster membership and mean 24-hour SBP, and that discrimination (c-index) may be similar between summary-variable models and cluster-label models. This is fine scientifically, but it means the manuscript should avoid implying improved prognostic performance. The contribution should be framed as automated integrative representation/visualization of ABPM level + variability rather than “added predictive information”.**

Our proposed approach can be compared with two different clinical approaches that are currently being used. In first instance, we compared the clusters against the office systolic blood pressure and risk scores such as SCORE2/SCORE2-OP. In this case, we indeed showed that our approach provided additional prognostic information as cluster 4 showed 55% higher CV risk compared to cluster 1 even after adjustment for both office systolic BP and SCORE2/SCORE-OP risk score. These findings were described in “24-Hour ABPM and incident CV events” section. Specifically, in lines 243-247 we described that:

*“Cluster 4 remained significant after adjustment for SCORE2/SCORE2-OP in a subset of 829 FLEMENGHO participants for whom risk scores could be calculated according to current guidelines. Compared with cluster 1 participants in cluster 4 had a 55% higher risk of incident CV events (HR: 1.55; 95% CI: 1.12–2.14; P=0.008; Supplemental Table ST3).”*

On the contrary, when we compared the derived clusters with summary indexes of 24-hour ABPM we indeed identified high collinearities. As such, we agree with the reviewer that this could only indicate that the two approaches are complementary. This has been extensively documented in the Discussion section. For example, in lines 314-321 we stated that the proposed approach is a complementary method of interpreting 24-hour ABPM recordings.

*“In this study, we have shown that multivariate ML algorithms might complement the current approach of clinical interpretation of 24-hour ABPM recordings. Our findings were consistent with current knowledge and showed that cluster 4 with the highest BP profiles had higher values in several BPV indices, as well as the highest CV risk. As shown in our study, CV outcomes were primarily associated with BP profiles but HR. Although clusters 1 and 2 demonstrated different HR profiles, there was no difference in CV risk between them. Of note, participants in cluster 2 reported higher intake of beta-blockers and other antihypertensive medication which can affect the HR.”*

Within the same context, in lines 327-340, we stated that with this study we aimed to evaluate the ability of unsupervised ML to automatically distinguish 24-hour ABPM with distinct CV risk profiles. Towards this direction, we acknowledged that the derived clusters (i.e. phenogroups) followed the already established knowledge regarding 24-hour ABPM.

*“It should be highlighted that our computational approach aimed to evaluate the ability of unsupervised ML models to automatically distinguish individuals into distinct risk groups. Towards this direction, the derived clusters fell well within the current BP classification. For example, cluster 1 profiles corresponded to normotensive individuals, clusters 2 and 3 displayed progressively elevated BP profiles, and cluster 4 represented mostly hypertensive individuals with markedly higher 24-hour ABPM values. In this study, we therefore propose a complementary approach to interpreting multivariate 24-hour ABPM recordings, one that facilitates rapid interpretation and visualization of individual 24-hour BP parameters together with BPV, while avoiding the need to calculate those multiple BPV coefficients separately. It also should be noted that major hypertensive guidelines do not provide formal cut-offs for BPV indexes, therefore they are not commonly used in clinical practice. With an ML-guided approach, we are able to integrate all key information derived from 24-hour ABPM time series data.”*

3. **Comment 3: Saying DTW can compare uneven-length series does not fully address the ABPM setting where daytime and nighttime have different sampling intervals (20 vs 45 minutes) and where DTW is typically index-based unless timestamps are incorporated. Please clarify explicitly whether time stamps are used, or whether the method treats measurements as equally spaced by index. If the latter, justify why this does not distort night segments, and add a brief sensitivity analysis (for example, resampling to a regular grid, or time-weighted distance).**

We would like to thank the reviewer for this insightful comment. In our analysis, DTW was applied to 24-hour ABPM time series indexed by measurement order. Consequently, timestamps were not used, and observations were treated as equally spaced. We included this clarification in “Model training” paragraph, lines 159-161, where we clearly describe: *“It should be noted that the timestamps were not included as a separate variable and thus the recordings were considered equally spaced by index.”*

We acknowledge that in the ABPM setting, daytime and nighttime measurements are acquired at different sampling intervals, which could lead to a relative underrepresentation of nighttime segments. However, the acquisition protocol was the same across all participants, which enables us to capture overall trajectory similarities (e.g. nocturnal dipping patterns). Towards this direction, clusters 3 and 4 revealed significant differences in nocturnal dipping in both systolic and diastolic BP (Figure 3 in main manuscript). Similarly, the centroids (Figure 2 in main manuscript) showed that clusters 3 and 4 had similar DBP profiles during daytime but they differentiated during nighttime. These findings can be considered strong indicators that DTW was able to capture BP patterns during nighttime.

However, to assess the impact of unequal sampling we conducted a sensitivity analysis in which all time series were resampled to a unified temporal axis. As Figure 1 below presents, CVI showed that the optimal number of clusters was 5 (instead of 4 in our original analysis).

**Figure 1**: Cluster validity index (CVI) for DTW combined with k-medoids on the resampled 24-hour ABPM recordings.

The derived phenogroups showed similar to our original analysis time series profiles. Cluster 5 had the highest 24-hour SBP and DBP profile followed by cluster 4. On the other hand, clusters 1 and 2 had similar low BP profiles resembling closely the relative similarities in our original analysis (Figure 2).

**Figure 2: Most representative profiles (medoids) per cluster as derived by the k-medoids algorithm trained on the resampled time-series FLEMENGHO dataset.**

However, using the resampled time series data and 5 clusters it seems that we are losing granularity over the BP variability indexes (Figure 3). Notably, all clusters showed similar nocturnal fall in SBP and DBP and only clusters 4 and 5 differentiated slightly in dispersion and weighted dispersion. Additionally, all clusters showed similar heart rate variability contrary to our original analysis which showed significant differences across clusters in all variability indexes in all three SBP, DBP and heart rate.

Figure 3: BP variability indexes of the derived clusters using the FLEMENGHO recordings resampled to equal length

Finally, evaluating the clinical significance of the derived clusters we observed similar to our original research patterns with cluster 5 showing the highest incidence rate of CV events. However, the rest of the clusters showed similar incident rates (Figure 4).

Figure 4: Incident of CV events per cluster derived by DTW combined with k-medoids used on the resampled 24-hour ABPM recordings.

**In conclusion, the results of our original research showed that the proposed model was able to identify patterns in nocturnal BP, while resampling to equal length and a unified temporal axis led to clusters with lower granularity in BPV**.
