# Supplementary material for: Machine learning–driven integration of 24-hour ambulatory blood pressure and its variability
Source: PLOS Digit Health. 2026 Jul 16;5(7):e0001499. doi: 10.1371/journal.pdig.0001499 (PMC13374967; doi:10.1371/journal.pdig.0001499)
Supplement: Attachment — Submitted filename: Editor_response_24052026_R4.docx [file pdig.0001499.s016.docx]

Reply to Editor:

1. **Comment 1: 1. Please amend your detailed Financial Disclosure statement. This is published with the article. It must therefore be completed in full sentences and contain the exact wording you wish to be published.**

**i. Please clarify all sources of financial support for your study. List the grants, grant numbers, and organizations that funded your study, including funding received from your institution. Please note that suppliers of material support, including research materials, should be recognized in the Acknowledgements section rather than in the Financial Disclosure.**

**ii. State the initials, alongside each funding source, of each author to receive each grant. For example: "This work was supported by the National Institutes of Health (####### to AM; ###### to CJ) and the National Science Foundation (###### to AM)."**

**iii. State what role the funders took in the study. If the funders had no role in your study, please state: “The funders had no role in study design, data collection and analysis, decision to publish, or preparation of the manuscript.”**

**iv. If any authors received a salary from any of your funders, please state which authors and which funders.**

We amended the financial statement and we clearly mentioned that the funders had no role in the study and/or analysis. Specifically, the adapted financial statement is:

*“The Research Unit Hypertension and Cardiovascular Epidemiology received grants from Internal Funds KU Leuven (C24M/21/025; DB/22/010/BM) and the Research Foundation Flanders (FWO grants: G0C5319N and G025625N). The funders had no role in study design, data collection and analysis, decision to publish, or preparation of the manuscript.”*

2. **Comment 2: Please provide an Author Summary. This should appear in your manuscript between the Abstract (if applicable) and the Introduction, and should be 150–200 words long. The aim should be to make your findings accessible to a wide audience that includes both scientists and non-scientists.**

We provided an author summary between abstract and Introduction as per journal’s requirements. Specifically, in lines 47-63 we summarized our study as follows:

*“Twenty-four hour ambulatory blood pressure monitoring (24-hour ABPM) is considered the best out-of-office blood pressure measurement to identify white-coat or masked hypertension. Current clinical practice relies mostly on analysing the average 24-hour blood pressure (BP) values or the average day-time and night-time values separately. At the same time, in literature, an abundance of blood pressure variability (BPV) has emerged as independent predictors of adverse cardiovascular events. However, the large number of BPV indices makes their interpretation and integration with the average 24-hour ABPM value more challenging. Therefore, we investigated if using a multivariable unsupervised machine learning model, we could derive clinically meaningful groups integrating both the average BP values and the BPV into a single model. By combining these features, we aimed to facilitate the use and interpretation of this otherwise complex time-series data. Using the raw 24-hour systolic and diastolic BP and heart rate time series recordings, we extracted 4 distinct profiles of 24-hour ABPM and BPV associated with different cardiovascular risk. Notably, our approach provided statistically significant additive information beyond the cardiovascular risk score that clinicians are currently using. Further testing our model in an external validation cohort showed similar results. Thus, time series clustering of ABPM recordings may facilitate the integrative interpretation of 24-hour ambulatory BP and BP variability”.*

3. **Comment 3: We have noticed that you have uploaded Supporting Information files, but you have not included a list of legends. Please add a full list of legends for your Supporting Information files after the references list.**

**lower granularity in BPV**.

We added the list of legends for the supporting material at the end of the main manuscript. Please see lines 506-538. We additionally adapted the Figures and Tables numbering to comply to journal’s regulations. Specifically, Figures are now referred as “Fig X” (instead of Figure X) while items in supporting materials are referred to as “Fig SX” and “Table SX” for figures and table respectively.

4. **Comment 4: Include a data access mechanism and address the reviewer's concerns regarding the use of DTW.**

We augmented the data availability statement by providing the contact details of the corresponding author, who can provide access to the used datasets. Specifically, in lines 373-375 we added:

*“For further information and to request access to the data please contact the corresponding author (prof. Tatiana Kuznetsova) via email (*[*tatiana.kouznetsova@med.kuleuven.be)*](mailto:tatiana.kouznetsova@med.kuleuven.be))*.”*

Additionally, we addressed the concern of the reviewer regarding the use of DTW in the limitation section where we clearly described the potential caveat of using DTW without timestamps and why this did not lead to under-representation of nighttime BP. In lines 349-354 we wrote:

*“Additionally, DTW treated ABPM measurements as equally spaced by index, which could lead to a relative underrepresentation of nighttime segments. However, the acquisition protocol was the same across all participants allowing us to capture trajectory similarities. Furthermore, our analysis showed significant differences in nocturnal dipping in both systolic and diastolic BP suggesting that the trained model captured nighttime BP patterns.”*
