# Supplementary material for: Machine learning–driven integration of 24-hour ambulatory blood pressure and its variability
Source: PLOS Digit Health. 2026 Jul 16;5(7):e0001499. doi: 10.1371/journal.pdig.0001499 (PMC13374967; doi:10.1371/journal.pdig.0001499)
Supplement: Peer review history

# Peer review: Decision Letter 0

**DOI:** 10.1371/journal.pdig.0001499.r001

**Authors:** Dhiya Al-Jumeily OBE

18 Dec 2025

PDIG-D-25-00920

Machine Learning–Driven Integration of 24-hour Ambulatory Blood Pressure and its Variability

PLOS Digital Health

Dear Dr. Kuznetsova,

Thank you for submitting your manuscript to PLOS Digital Health. After careful consideration, we feel that it has merit but does not fully meet PLOS Digital Health's publication criteria as it currently stands. Therefore, we invite you to submit a revised version of the manuscript that addresses the points raised during the review process.

Please submit your revised manuscript by Feb 16 2026 11:59PM. If you will need more time than this to complete your revisions, please reply to this message or contact the journal office at digitalhealth@plos.org. When you're ready to submit your revision, log on to https://www.editorialmanager.com/pdig/ and select the 'Submissions Needing Revision' folder to locate your manuscript file.

Please include the following items when submitting your revised manuscript:

* A letter that responds to each point raised by the editor and reviewer(s). You should upload this letter as a separate file labeled 'Response to Reviewers'. This file does not need to include responses to any formatting updates and technical items listed in the 'Journal Requirements' section below.

* A marked-up copy of your manuscript that highlights changes made to the original version. You should upload this as a separate file labeled 'Revised Manuscript with Track Changes'.

* An unmarked version of your revised paper without tracked changes. You should upload this as a separate file labeled 'Manuscript'.

If you would like to make changes to your financial disclosure, competing interests statement, or data availability statement, please make these updates within the submission form at the time of resubmission. Guidelines for resubmitting your figure files are available below the reviewer comments at the end of this letter.

We look forward to receiving your revised manuscript.

Kind regards,

Dhiya Al-Jumeily OBE, PhD

Section Editor

PLOS Digital Health

Leo Anthony Celi

Editor-in-Chief

PLOS Digital Health

orcid.org/0000-0001-6712-6626

**Journal Requirements:**

1. We have amended your Competing Interest statement to comply with journal style. We kindly ask that you double check the statement and let us know if anything is incorrect.

2. Please amend your detailed Financial Disclosure statement. This is published with the article. It must therefore be completed in full sentences and contain the exact wording you wish to be published.

1. Please clarify all sources of funding (financial or material support) for your study. List the grants (with grant number) or organizations (with url) that supported your study, including funding received from your institution.

2. State the initials, alongside each funding source, of each author to receive each grant.

3. State what role the funders took in the study. If the funders had no role in your study, please state: “The funders had no role in study design, data collection and analysis, decision to publish, or preparation of the manuscript.”

4. If any authors received a salary from any of your funders, please state which authors and which funders.

3. In the online submission form, you indicated that The data underlying this article will be shared on reasonable request to the corresponding author.

All PLOS journals now require all data underlying the findings described in their manuscript to be freely available to other researchers, either

1. In a public repository,

2. Within the manuscript itself, or

3. Uploaded as supplementary information.

This policy applies to all data except where public deposition would breach compliance with the protocol approved by your research ethics board. If your data cannot be made publicly available for ethical or legal reasons (e.g., public availability would compromise patient privacy), please explain your reasons by return email and your exemption request will be escalated to the editor for approval. Your exemption request will be handled independently and will not hold up the peer review process, but will need to be resolved should your manuscript be accepted for publication. One of the Editorial team will then be in touch if there are any issues.

4. Please insert an Ethics Statement at the beginning of your Methods section, under a subheading 'Ethics Statement'. It must include:

1) The name(s) of the Institutional Review Board(s) or Ethics Committee(s)

2) The approval number(s), or a statement that approval was granted by the named board(s)

5. We have noticed that you have uploaded Supporting Information files, but you have not included a list of legends. Please add a full list of legends for your Supporting Information files after the references list.

6. If the reviewer comments include a recommendation to cite specific previously published works, please review and evaluate these publications to determine whether they are relevant and should be cited. There is no requirement to cite these works unless the editor has indicated otherwise.

[Note: HTML markup is below. Please do not edit.]

**Reviewers' Comments:**

Reviewer's Responses to Questions

**Comments to the Author**

1. Does this manuscript meet PLOS Digital Health’s publication criteria? Is the manuscript technically sound, and do the data support the conclusions? The manuscript must describe methodologically and ethically rigorous research with conclusions that are appropriately drawn based on the data presented.

Reviewer #1: Partly

Reviewer #2: Yes

Reviewer #3: No

**********

2. Has the statistical analysis been performed appropriately and rigorously?

Reviewer #1: Yes

Reviewer #2: Yes

Reviewer #3: No

**********

3. Have the authors made all data underlying the findings in their manuscript fully available (please refer to the Data Availability Statement at the start of the manuscript PDF file)?

The PLOS Data policy requires authors to make all data underlying the findings described in their manuscript fully available without restriction, with rare exception. The data should be provided as part of the manuscript or its supporting information, or deposited to a public repository. For example, in addition to summary statistics, the data points behind means, medians and variance measures should be available. If there are restrictions on publicly sharing data—e.g. participant privacy or use of data from a third party—those must be specified.

Reviewer #1: No

Reviewer #2: Yes

Reviewer #3: No

**********

4. Is the manuscript presented in an intelligible fashion and written in standard English?

PLOS Digital Health does not copyedit accepted manuscripts, so the language in submitted articles must be clear, correct, and unambiguous. Any typographical or grammatical errors should be corrected at revision, so please note any specific errors here.

Reviewer #1: Yes

Reviewer #2: Yes

Reviewer #3: Yes

**********

5. Review Comments to the Author

Please use the space provided to explain your answers to the questions above. You may also include additional comments for the author, including concerns about dual publication, research ethics, or publication ethics. (Please upload your review as an attachment if it exceeds 20,000 characters)

Reviewer #1: Major comments:

1. Cluster 4 clearly has the highest 24‑h SBP/DBP and the highest BPV, and also the highest event rate. However, the Cox models adjust for office SBP and standard risk factors, but not for mean 24‑h SBP (or day/night SBP or simple dipping pattern). The Discussion and Author Summary imply added information beyond existing ABPM interpretation and beyond “24‑hour ambulatory SBP”. To support this key claim, please: (i) add models including mean 24‑h SBP (and/or conventional ABPM categories) and then cluster membership, and report whether clusters still predict events; (ii) Ideally, compare model performance (C‑statistics, IDI/NRI or likelihood ratio tests) between: (a) office risk factors, (b) office + mean 24‑h SBP (±dipping/BPV), and (c) office + ABPM summaries + cluster membership; and (iii) clarify the wording throughout if the clustering does *not* provide independent information beyond mean 24‑h SBP.

2. You state that raw ABPM recordings were used “without any signal processing” and that k‑medoids with dynamic time warping was applied to SBP, DBP and HR time series. Please clarify:

+ How irregular sampling (20‑min daytime, 45‑min night) and occasional missing readings were handled in the DTW distance (regular resampling? inclusion of timestamps? simple sequence index?).

+ Whether SBP, DBP and HR were scaled/standardized before computing multivariate DTW.

+ The exact multivariate DTW implementation (e.g., independent DTW per channel averaged vs joint multivariate DTW) and package used.

+ Provide at least a brief assessment of cluster stability (e.g., bootstrapping or Jaccard similarity) and a sensitivity analysis to different numbers of clusters (k=3,5) beyond the single hybrid CVI curve on page 30.

3. In EPOGH, it is stated that the “trained model” was applied and four clusters were again observed. Please specify clearly whether:

+ The FLEMENGHO medoids were fixed and each EPOGH subject was assigned to the nearest existing medoid, or clustering was re‑run de novo in EPOGH.

+ If medoids were reused, describe any distributional differences between cohorts and whether distances to medoids were similar. If clustering was repeated, then it is not a strict external validation of the *same* unsupervised structure and this should be acknowledged.

4. Clusters 1 and 2 have very similar SBP/DBP profiles but differ mainly in HR; clusters 3 and 4 show higher BP and BPV. It would help readers if you:

+ Provide clearer clinical labels/interpretation (e.g., “normotensive/high HR”, “intermediate BP/high dipping”, “hypertensive/high variability”).

+ Comment more explicitly on why HR‑driven differentiation between clusters 1 and 2 does not appear to translate into clearly different risk, and whether HR adds anything once BP level is accounted for.

+ Consider a model where cluster membership is replaced by continuous mean 24‑h BP and HR (±BPV indices) to show what additional information the clustering itself contributes.

5. The current Data Availability statement (“data will be shared on reasonable request”) does not meet PLOS’ requirement for open data in a public repository.

Minor comments:

1. Use one term consistently for groups (“clusters” vs “phenogroups”). Currently both are used somewhat interchangeably. Standardize “24‑hour” vs “24-h” across abstract, text and figures.

2. You describe comparing continuous variables with the Z distribution and proportions with χ². It may be clearer to state that you used linear/logistic regression or ANOVA/χ² tests as appropriate. Specify how proportional hazards assumptions were checked in the Cox models.

3. Radar plots in Figures 3 and 6 are visually appealing but dense; consider adding a short explanation in the legends of what a positive vs negative Z‑score means relative to the overall cohort and perhaps including one or two key BPV indices in a simpler bar plot in the main text. Please ensure all abbreviations used in Tables 1–3 are defined in the footnotes at first appearance (e.g., “ARV”, “CoV”).

4. The Limitations section could more clearly state that the cohorts are European, with limited ethnic diversity, and that generalizability to other populations, and to contemporary ABPM/wearable devices, remains to be tested.

Reviewer #2: Please write about the unsupervised ML algorithm you used to cluster datasets. why you have chosen the k-medoids clustering algorithm. Have you compared your choice of algorithm with other algorithms in the unsupervised ML domain? What is the speciality of the K-medoids clustering algorithm?

Reviewer #3: Thank you for the opportunity to review this manuscript. The topic—leveraging machine learning to integrate 24-hour ABPM and BP variability—is relevant and aligns with the scope of digital health research. However, several major issues limit the scientific contribution and prevent the manuscript from meeting publication standards in its current form.

1. Limited novelty and incremental insight

The unsupervised clustering approach (DTW + k-medoids) applies established methods without demonstrating clear methodological innovation. The resulting four phenogroups largely recapitulate known BP categories (normotensive → hypertensive), as reflected in the ABPM profiles and cluster characteristics (Tables 1–2; Figures 1–2) . The study does not clearly show what new insights are gained beyond conventional ABPM summaries or standard BPV metrics.

2. Statistical analysis requires substantial strengthening

Key analytical concerns include:

Absence of cluster stability assessments or sensitivity analyses;

No evaluation of model calibration, discrimination, or predictive improvement (e.g., C-index, NRI);

Limited adjustment for important confounders;

Unclear testing of proportional hazards assumptions;

Insufficient external validation, with primarily descriptive comparisons (Figure 5–6) .

Overall, the evidence supporting the clusters’ independent prognostic value remains incomplete.

3. Data availability does not meet PLOS requirements

The statement that data “will be shared upon reasonable request” does not comply with PLOS’s open data policy. Raw ABPM time series, cluster assignments, and analysis code must be publicly accessible.

4. Interpretation and clinical implications need clearer articulation

Although the manuscript proposes integrative interpretation of ABPM + BPV, the clinical meaning of each cluster remains insufficiently differentiated. Differences between clusters largely reflect BP level rather than novel temporal or variability phenotypes (Figures 2–3) . More explicit justification for clinical utility is needed.

5. Presentation and methodological clarity

The manuscript is generally well written, but several methodological choices—such as handling of heterogeneous sampling intervals, rationale for DTW parameters, and pre-processing decisions—require clearer explanation.

While the work addresses an important problem, substantial revisions are necessary to enhance methodological robustness, demonstrate true incremental value, and comply with data-sharing requirements. I encourage the authors to strengthen the analytical framework, expand validation, and more clearly define the clinical significance of the proposed phenogroups.

**********

6. PLOS authors have the option to publish the peer review history of their article (what does this mean?). If published, this will include your full peer review and any attached files.

**Do you want your identity to be public for this peer review?**   If you choose “no”, your identity will remain anonymous but your review may still be made public.

For information about this choice, including consent withdrawal, please see our Privacy Policy.

Reviewer #1: No

Reviewer #2: **Yes:**  Pethuru Raj Chelliah

Reviewer #3: No

**********

[NOTE: If reviewer comments were submitted as an attachment file, they will be attached to this email and accessible via the submission site. Please log into your account, locate the manuscript record, and check for the action link "View Attachments". If this link does not appear, there are no attachment files.]

**Figure resubmission:**

While revising your submission, we strongly recommend that you use PLOS’s NAAS tool (https://ngplosjournals.pagemajik.ai/artanalysis) to test your figure files. NAAS can convert your figure files to the TIFF file type and meet basic requirements (such as print size, resolution), or provide you with a report on issues that do not meet our requirements and that NAAS cannot fix.

After uploading your figures to PLOS’s NAAS tool - https://ngplosjournals.pagemajik.ai/artanalysis, NAAS will process the files provided and display the results in the "Uploaded Files" section of the page as the processing is complete. If the uploaded figures meet our requirements (or NAAS is able to fix the files to meet our requirements), the figure will be marked as "fixed" above. If NAAS is unable to fix the files, a red "failed" label will appear above. When NAAS has confirmed that the figure files meet our requirements, please download the file via the download option, and include these NAAS processed figure files when submitting your revised manuscript.

**Reproducibility:**

To enhance the reproducibility of your results, we recommend that authors of applicable studies deposit laboratory protocols in protocols.io, where a protocol can be assigned its own identifier (DOI) such that it can be cited independently in the future. Additionally, PLOS ONE offers an option to publish peer-reviewed clinical study protocols. Read more information on sharing protocols at https://plos.org/protocols?utm_medium=editorial-email&utm_source=authorletters&utm_campaign=protocols

# Author comment: Author response to Decision Letter 1

**DOI:** 10.1371/journal.pdig.0001499.r002

16 Feb 2026

Attachment Submitted filename: Reviewer comments_14022026.docx

# Peer review: Decision Letter 1

**DOI:** 10.1371/journal.pdig.0001499.r003

**Authors:** Dhiya Al-Jumeily OBE, Patrick Wai-Hang Kwong

12 Mar 2026

PDIG-D-25-00920R1

Machine Learning–Driven Integration of 24-hour Ambulatory Blood Pressure and its Variability

PLOS Digital Health

Dear Dr. Kuznetsova,

Thank you for submitting your manuscript to PLOS Digital Health. After careful consideration, we feel that it has merit but does not fully meet PLOS Digital Health's publication criteria as it currently stands. Therefore, we invite you to submit a revised version of the manuscript that addresses the points raised during the review process.

Please submit your revised manuscript by May 11 2026 11:59PM. If you will need more time than this to complete your revisions, please reply to this message or contact the journal office at digitalhealth@plos.org. When you're ready to submit your revision, log on to https://www.editorialmanager.com/pdig/ and select the 'Submissions Needing Revision' folder to locate your manuscript file.

Please include the following items when submitting your revised manuscript:

* A letter that responds to each point raised by the editor and reviewer(s). You should upload this letter as a separate file labeled 'Response to Reviewers'. This file does not need to include responses to any formatting updates and technical items listed in the 'Journal Requirements' section below.

* A marked-up copy of your manuscript that highlights changes made to the original version. You should upload this as a separate file labeled 'Revised Manuscript with Track Changes'.

* An unmarked version of your revised paper without tracked changes. You should upload this as a separate file labeled 'Manuscript'.

If you would like to make changes to your financial disclosure, competing interests statement, or data availability statement, please make these updates within the submission form at the time of resubmission. Guidelines for resubmitting your figure files are available below the reviewer comments at the end of this letter.

We look forward to receiving your revised manuscript.

Kind regards,

Patrick Wai-Hang Kwong

Academic Editor

PLOS Digital Health

Dhiya Al-Jumeily OBE

Section Editor

PLOS Digital Health

Leo Anthony Celi

Editor-in-Chief

PLOS Digital Health

orcid.org/0000-0001-6712-6626

**Journal Requirements:**

If the reviewer comments include a recommendation to cite specific previously published works, please review and evaluate these publications to determine whether they are relevant and should be cited. There is no requirement to cite these works unless the editor has indicated otherwise.

**Additional Editor Comments (if provided):**

Please address the remaining concerns raised by the reviewer regarding the study implications and the use of DTW, particularly the issue related to differences in the sample timeframes.

[Note: HTML markup is below. Please do not edit.]

**Reviewers' Comments:**

Reviewer's Responses to Questions

**Comments to the Author**

1. If the authors have adequately addressed your comments raised in a previous round of review and you feel that this manuscript is now acceptable for publication, you may indicate that here to bypass the “Comments to the Author” section, enter your conflict of interest statement in the “Confidential to Editor” section, and submit your "Accept" recommendation.

Reviewer #1: (No Response)

Reviewer #2: All comments have been addressed

**********

2. Does this manuscript meet PLOS Digital Health’s publication criteria? Is the manuscript technically sound, and do the data support the conclusions? The manuscript must describe methodologically and ethically rigorous research with conclusions that are appropriately drawn based on the data presented.

Reviewer #1: Yes

Reviewer #2: Yes

**********

3. Has the statistical analysis been performed appropriately and rigorously?

Reviewer #1: Yes

Reviewer #2: Yes

**********

4. Have the authors made all data underlying the findings in their manuscript fully available (please refer to the Data Availability Statement at the start of the manuscript PDF file)?

The PLOS Data policy requires authors to make all data underlying the findings described in their manuscript fully available without restriction, with rare exception. The data should be provided as part of the manuscript or its supporting information, or deposited to a public repository. For example, in addition to summary statistics, the data points behind means, medians and variance measures should be available. If there are restrictions on publicly sharing data—e.g. participant privacy or use of data from a third party—those must be specified.

Reviewer #1: No

Reviewer #2: Yes

**********

5. Is the manuscript presented in an intelligible fashion and written in standard English?

PLOS Digital Health does not copyedit accepted manuscripts, so the language in submitted articles must be clear, correct, and unambiguous. Any typographical or grammatical errors should be corrected at revision, so please note any specific errors here.

Reviewer #1: Yes

Reviewer #2: Yes

**********

6. Review Comments to the Author

Please use the space provided to explain your answers to the questions above. You may also include additional comments for the author, including concerns about dual publication, research ethics, or publication ethics. (Please upload your review as an attachment if it exceeds 20,000 characters)

**Reviewer #1:**   The revision is substantially improved. The manuscript is clearer on (i) how DTW+k-medoids was implemented (including the library), (ii) how the external cohort was used (medoid assignment rather than re-clustering), and (iii) cluster stability and basic sensitivity to k. The clinical interpretation of clusters is also sharper. That said, two core issues remain before the work is publishable:

1. The current approach (“shared on reasonable request” and ethics approval needed) is unlikely to meet PLOS requirements as written. PLOS Digital Health requires that data needed to replicate findings be publicly available at publication, and when legal/ethical constraints apply, the statement must specify where the data reside and the exact access mechanism.

2. The added analyses (as described in the response) suggest strong collinearity between cluster membership and mean 24-hour SBP, and that discrimination (c-index) may be similar between summary-variable models and cluster-label models. This is fine scientifically, but it means the manuscript should avoid implying improved prognostic performance. The contribution should be framed as automated integrative representation/visualization of ABPM level + variability rather than “added predictive information”.

3. Saying DTW can compare uneven-length series does not fully address the ABPM setting where daytime and nighttime have different sampling intervals (20 vs 45 minutes) and where DTW is typically index-based unless timestamps are incorporated. Please clarify explicitly whether time stamps are used, or whether the method treats measurements as equally spaced by index. If the latter, justify why this does not distort night segments, and add a brief sensitivity analysis (for example, resampling to a regular grid, or time-weighted distance).

**Reviewer #2:**   You have meticulously addressed all the comments of various reviewers

**********

7. PLOS authors have the option to publish the peer review history of their article (what does this mean?). If published, this will include your full peer review and any attached files.

**Do you want your identity to be public for this peer review?**   If you choose “no”, your identity will remain anonymous but your review may still be made public.

For information about this choice, including consent withdrawal, please see our Privacy Policy.

Reviewer #1: No

Reviewer #2: **Yes:**  Pethuru Raj Chelliah

**********

[NOTE: If reviewer comments were submitted as an attachment file, they will be attached to this email and accessible via the submission site. Please log into your account, locate the manuscript record, and check for the action link "View Attachments". If this link does not appear, there are no attachment files.]

**Figure resubmission:**

While revising your submission, we strongly recommend that you use PLOS’s NAAS tool (https://ngplosjournals.pagemajik.ai/artanalysis) to test your figure files. NAAS can convert your figure files to the TIFF file type and meet basic requirements (such as print size, resolution), or provide you with a report on issues that do not meet our requirements and that NAAS cannot fix.

After uploading your figures to PLOS’s NAAS tool - https://ngplosjournals.pagemajik.ai/artanalysis, NAAS will process the files provided and display the results in the "Uploaded Files" section of the page as the processing is complete. If the uploaded figures meet our requirements (or NAAS is able to fix the files to meet our requirements), the figure will be marked as "fixed" above. If NAAS is unable to fix the files, a red "failed" label will appear above. When NAAS has confirmed that the figure files meet our requirements, please download the file via the download option, and include these NAAS processed figure files when submitting your revised manuscript.

**Reproducibility:**

To enhance the reproducibility of your results, we recommend that authors of applicable studies deposit laboratory protocols in protocols.io, where a protocol can be assigned its own identifier (DOI) such that it can be cited independently in the future. Additionally, PLOS ONE offers an option to publish peer-reviewed clinical study protocols. Read more information on sharing protocols at https://plos.org/protocols?utm_medium=editorial-email&utm_source=authorletters&utm_campaign=protocols

# Author comment: Author response to Decision Letter 2

**DOI:** 10.1371/journal.pdig.0001499.r004

30 Apr 2026

Attachment Submitted filename: Reviewers_response_30042026_R3.docx

# Editor report: Decision Letter 2

**DOI:** 10.1371/journal.pdig.0001499.r005

**Authors:** Dhiya Al-Jumeily OBE, Patrick Wai-Hang Kwong

13 May 2026

PDIG-D-25-00920R2

Machine Learning–Driven Integration of 24-hour Ambulatory Blood Pressure and its Variability

PLOS Digital Health

Dear Dr. Kuznetsova,

Thank you for submitting your manuscript to PLOS Digital Health. After careful consideration, we feel that it has merit but does not fully meet PLOS Digital Health's publication criteria as it currently stands. Therefore, we invite you to submit a revised version of the manuscript that addresses the points raised during the review process.

Please submit your revised manuscript by Jun 12 2026 11:59PM. If you will need more time than this to complete your revisions, please reply to this message or contact the journal office at digitalhealth@plos.org. When you're ready to submit your revision, log on to https://www.editorialmanager.com/pdig/ and select the 'Submissions Needing Revision' folder to locate your manuscript file.

Please include the following items when submitting your revised manuscript:

* A letter that responds to each point raised by the editor and reviewer(s). You should upload this letter as a separate file labeled 'Response to Reviewers'. This file does not need to include responses to any formatting updates and technical items listed in the 'Journal Requirements' section below.

* A marked-up copy of your manuscript that highlights changes made to the original version. You should upload this as a separate file labeled 'Revised Manuscript with Track Changes'.

* An unmarked version of your revised paper without tracked changes. You should upload this as a separate file labeled 'Manuscript'.

If you would like to make changes to your financial disclosure, competing interests statement, or data availability statement, please make these updates within the submission form at the time of resubmission. Guidelines for resubmitting your figure files are available below the reviewer comments at the end of this letter.

As the corresponding author, your ORCID iD is verified in the submission system and will appear in the published article. PLOS supports the use of ORCID, and we encourage all coauthors to register for an ORCID iD and use it as well. Please encourage your coauthors to verify their ORCID iD within the submission system before final acceptance, as unverified ORCID iDs will not appear in the published article. Only the individual author can complete the verification step; PLOS staff cannot verify ORCID iDs on behalf of authors.

We look forward to receiving your revised manuscript.

Kind regards,

Dhiya Al-Jumeily OBE, PhD

Section Editor

PLOS Digital Health

Patrick Wai-Hang Kwong

Academic Editor

PLOS Digital Health

Leo Anthony Celi

Editor-in-Chief

PLOS Digital Health

orcid.org/0000-0001-6712-6626

**Journal Requirements:**

1. Please amend your detailed Financial Disclosure statement. This is published with the article. It must therefore be completed in full sentences and contain the exact wording you wish to be published.

i. Please clarify all sources of financial support for your study. List the grants, grant numbers, and organizations that funded your study, including funding received from your institution. Please note that suppliers of material support, including research materials, should be recognized in the Acknowledgements section rather than in the Financial Disclosure.

ii. State the initials, alongside each funding source, of each author to receive each grant. For example: "This work was supported by the National Institutes of Health (####### to AM; ###### to CJ) and the National Science Foundation (###### to AM)."

iii. State what role the funders took in the study. If the funders had no role in your study, please state: “The funders had no role in study design, data collection and analysis, decision to publish, or preparation of the manuscript.”

iv. If any authors received a salary from any of your funders, please state which authors and which funders.

2. Please provide an Author Summary. This should appear in your manuscript between the Abstract (if applicable) and the Introduction, and should be 150–200 words long. The aim should be to make your findings accessible to a wide audience that includes both scientists and non-scientists. Sample summaries can be found on our website under Submission Guidelines:

https://journals.plos.org/digitalhealth/s/submission-guidelines#loc-parts-of-a-submission

3. We have noticed that you have uploaded Supporting Information files, but you have not included a list of legends. Please add a full list of legends for your Supporting Information files after the references list.

If the reviewer comments include a recommendation to cite specific previously published works, please review and evaluate these publications to determine whether they are relevant and should be cited. There is no requirement to cite these works unless the editor has indicated otherwise.

**Additional Editor Comments (if provided):**

Please include a data access mechanism and address the reviewer's concerns regarding the use of DTW.

[Note: HTML markup is below. Please do not edit.]

**Reviewers' Comments:**

[NOTE: If reviewer comments were submitted as an attachment file, they will be attached to this email and accessible via the submission site. Please log into your account, locate the manuscript record, and check for the action link "View Attachments". If this link does not appear, there are no attachment files.]

**Figure resubmission:**

While revising your submission, we strongly recommend that you use PLOS’s NAAS tool (https://ngplosjournals.pagemajik.ai/artanalysis) to test your figure files. NAAS can convert your figure files to the TIFF file type and meet basic requirements (such as print size, resolution), or provide you with a report on issues that do not meet our requirements and that NAAS cannot fix.

After uploading your figures to PLOS’s NAAS tool - https://ngplosjournals.pagemajik.ai/artanalysis, NAAS will process the files provided and display the results in the "Uploaded Files" section of the page as the processing is complete. If the uploaded figures meet our requirements (or NAAS is able to fix the files to meet our requirements), the figure will be marked as "fixed" above. If NAAS is unable to fix the files, a red "failed" label will appear above. When NAAS has confirmed that the figure files meet our requirements, please download the file via the download option, and include these NAAS processed figure files when submitting your revised manuscript.

**Reproducibility:**

To enhance the reproducibility of your results, we recommend that authors of applicable studies deposit laboratory protocols in protocols.io, where a protocol can be assigned its own identifier (DOI) such that it can be cited independently in the future. Additionally, PLOS ONE offers an option to publish peer-reviewed clinical study protocols. Read more information on sharing protocols at https://plos.org/protocols?utm_medium=editorial-email&utm_source=authorletters&utm_campaign=protocols

# Author comment: Author response to Decision Letter 3

**DOI:** 10.1371/journal.pdig.0001499.r006

28 May 2026

Attachment Submitted filename: Editor_response_24052026_R4.docx

# Editor report: Decision Letter 3

**DOI:** 10.1371/journal.pdig.0001499.r007

**Authors:** Dhiya Al-Jumeily OBE, Patrick Wai-Hang Kwong

29 May 2026

Machine Learning–Driven Integration of 24-hour Ambulatory Blood Pressure and its Variability

PDIG-D-25-00920R3

Dear Dr. Kuznetsova,

We are pleased to inform you that your manuscript 'Machine Learning–Driven Integration of 24-hour Ambulatory Blood Pressure and its Variability' has been provisionally accepted for publication in PLOS Digital Health.

Before your manuscript can be formally accepted you will need to complete some formatting changes, which you will receive in a follow-up email from a member of our team.

Please note that your manuscript will not be scheduled for publication until you have made the required changes, so a swift response is appreciated.

IMPORTANT: The editorial review process is now complete. PLOS will only permit corrections to spelling, formatting or significant scientific errors from this point onwards. Requests for major changes, or any which affect the scientific understanding of your work, will cause delays to the publication date of your manuscript.

If your institution or institutions have a press office, please notify them about your upcoming paper to help maximize its impact. If they'll be preparing press materials, please inform our press team as soon as possible -- no later than 48 hours after receiving the formal acceptance. Your manuscript will remain under strict press embargo until 2 pm Eastern Time on the date of publication. For more information, please contact digitalhealth@plos.org.

Thank you again for supporting Open Access publishing; we are looking forward to publishing your work in PLOS Digital Health.

Best regards,

Dhiya Al-Jumeily OBE, PhD

Section Editor

PLOS Digital Health

***********************************************************

**Additional Editor Comments (if provided):**

**Reviewer Comments (if any, and for reference):**
